# NOTCH1, HIF1A and Other Cancer-Related Proteins in Lung Tissue from Uranium Miners—Variation by Occupational Exposure and Subtype of Lung Cancer

**DOI:** 10.1371/journal.pone.0045305

**Published:** 2012-09-17

**Authors:** Beate Pesch, Swaantje Casjens, Ingo Stricker, Daniela Westerwick, Dirk Taeger, Sylvia Rabstein, Thorsten Wiethege, Andrea Tannapfel, Thomas Brüning, Georg Johnen

**Affiliations:** 1 Institute for Prevention and Occupational Medicine of the German Social Accident Insurance, Institute of the Ruhr University Bochum (IPA), Bochum, Germany; 2 Institute of Pathology, Ruhr University Bochum, Bochum, Germany; University of Porto, Portugal

## Abstract

**Background:**

Radon and arsenic are established pulmonary carcinogens. We investigated the association of cumulative exposure to these carcinogens with NOTCH1, HIF1A and other cancer-specific proteins in lung tissue from uranium miners.

**Methodology/Principal Findings:**

Paraffin-embedded tissue of 147 miners was randomly selected from an autopsy repository by type of lung tissue, comprising adenocarcinoma (AdCa), squamous cell carcinoma (SqCC), small cell lung cancer (SCLC), and cancer-free tissue. Within each stratum, we additionally stratified by low or high level of exposure to radon or arsenic. Lifetime exposure to radon and arsenic was estimated using a quantitative job-exposure matrix developed for uranium mining. For 22 cancer-related proteins, immunohistochemical scores were calculated from the intensity and percentage of stained cells. We explored the associations of these scores with cumulative exposure to radon and arsenic with Spearman rank correlation coefficients (r_s_). Occupational exposure was associated with an up-regulation of NOTCH1 (radon r_s_ = 0.18, 95% CI 0.02–0.33; arsenic: r_s_ = 0.23, 95% CI 0.07–0.38). Moreover, we investigated whether these cancer-related proteins can classify lung cancer using supervised and unsupervised classification. MUC1 classified lung cancer from cancer-free tissue with a failure rate of 2.1%. A two-protein signature discriminated SCLC (HIF1A low), AdCa (NKX2-1 high), and SqCC (NKX2-1 low) with a failure rate of 8.4%.

**Conclusions/Significance:**

These results suggest that the radiation-sensitive protein NOTCH1 can be up-regulated in lung tissue from uranium miners by level of exposure to pulmonary carcinogens. We evaluated a three-protein signature consisting of a physiological protein (MUC1), a cancer-specific protein (HIF1A), and a lineage-specific protein (NKX2-1) that could discriminate lung cancer and its major subtypes with a low failure rate.

## Introduction

In East Germany, extensive uranium mining was undertaken for the Soviet nuclear industry from 1946 until 1990 [1]. Poor working conditions in the so-called WISMUT mining company led to very high levels of exposure to ionizing radiation [2]. Exposure to arsenic occurred in some mines depending on the metal content of the ore.

A comprehensive job-exposure matrix (JEM) was developed for the quantitative assessment of exposure to radon, arsenic, and quartz dust based on extensive measurements [3]. The largest single cohort of uranium miners was established showing a dose-dependent excess risk of lung cancer by radon exposure [4], [5].

Biological research on radiation-induced carcinogenesis has been focussed on the damage of the genome. So far, available results do not consistently suggest a radon-specific mutation of *TP53* [6]. However, little is known about other genes and whether exposure to radiation can be related to cancer-specific proteins: Thyroid cancers from the Chernobyl tissue repository have been examined in order to detect radiation-specific protein signatures [7], [8], and radiation has been associated with *NOTCH1* mutations in the development of lymphomas [9].

It could be hypothesized that radiation acts on genes that are prone to instability and activated in cancer-associated pathways like *NOTCH1*. We took advantage of a unique tissue repository of WISMUT miners that had been opened for research after German reunification [10] to explore protein patterns in lung tissue. The statistical analysis of these data revealed a shift towards small cell lung cancer (SCLC) and squamous cell carcinoma (SqCC) at the expense of adenocarcinoma (AdCa) with increasing exposure to radon or arsenic [11], [12]. An even stronger shift by level of exposure was observed for smoking in a large pooled analysis of lung cancer studies [13]. Here, we continue our research on the observed exposure-related shifts in the distribution of subtypes of lung cancer by exploring protein patterns. SCLC and SqCC appear to exhibit a higher ‘stemness’ than AC following a larger damage of the lung architecture. We therefore hypothesize that candidate proteins associated with stemness are more frequently expressed in SCLC and SqCC than in AdCa or cancer-free lung tissue. In particular, we investigate 1) whether we can detect an association of occupational exposure to radon or arsenic with the expression of candidate proteins, 2) if we can discriminate the subtypes of lung cancer with these proteins, and 3) if influences of exposure add to the discrimination of the major subtypes of lung cancer.

## Materials and Methods

### Study Design

A quantitative job-exposure matrix was applied to the occupational data of uranium miners with lung tissue in the WISMUT autopsy repository. Cumulative radon exposure was given in working level months (WLM) as previously described [12]. We stratified by >1000 WLM for high and by <500 WLM for low radon exposure. Cumulative airborne arsenic exposure was assessed as described earlier [11] and classified as high by >100 µg/m^3^ years and as low by <50 µg/m^3^ years. The cut-offs were based on the distribution of the exposure variables in miners with archived lung tissue samples and on the availability of tissue samples for rare combinations, like low radon and high arsenic exposure.

We searched the WISMUT autopsy repository for available lung tissue samples of uranium miners with a marked contrast in exposure to radon and arsenic. Ten male miners each were randomly selected from the database of the repository within 16 strata of an orthogonal study design. Stratification was performed by combinations of low or high exposure to radon and arsenic within four groups of 40 miners each, either cancer-free or with AdCa, SqCC, or SCLC. Information on silicosis, lung cancer, and occupational exposure were extracted from the database of the archive [14]. Smoking status could be classified as ever or never for 119 miners from employment records and medical documents of the WISMUT archives. This research, including ethical issues, with historical tissue samples from deceased uranium miners was authorized by the German government in a direct treaty with our institutes signed July 29, 2003. The study was based on anonymous tissue samples and conducted according to the principles expressed in the Declaration of Helsinki.

Three German pathologists re-classified lung cancer of archived tissue according to the WHO classification [15], [16]. We retrieved samples where at least two of the pathologists were in agreement and excluded mixed forms for improvement of classification. Samples were available for 146 out of 160 subjects randomly ascertained from the database. An additional set of 15 samples was selected for validation. A blind reading of the newly generated slides (by I.S. and D.W.) confirmed the former histological classifications.

### Immunohistochemical Analysis

Due to the prevalence of tuberculosis in miners tissue specimens underwent long-term fixation in formalin. The degradation of RNA by formalin prevented a search for RNA expression signatures by screening techniques like DNA microarrays. However, initial experiments indicated that immunohistochemistry was still applicable for many samples [17]. For that reason, we selected 30 proteins from the literature as possibly associated with lung cancer (e.g., EGFR, NKX2-1), lung development and lineage determination (e.g., NOTCH1), lung physiology (e.g., SFTPC, MUC1), tissue remodeling following exposure to radon (e.g., MMP2), or the preference of arsenic for cytokeratins (KRT5, KRT14). Immunohistochemical assays could be established for 22 proteins (supplemental **Table S1**). For AKT1, ATM, CDKN2A, ERCC2, ILK, NFKB1, PTEN, and WIF staining could not be established with the archived material. Tissue microarrays (TMAs) could not be employed due to the unusual mechanical properties of the WISMUT paraffin material, which was very brittle and not suitable for the punching tubes used with the TMA machine. Therefore, sections of 4 µm were cut from individual formalin-fixed paraffin-embedded samples and mounted on aminopropyltriethoxysilane slides. All slides were deparaffinized and rehydrated in graded alcohols (100%, 96%, and 70%). Antigen retrieval for immunostaining was performed by heating the samples in citrate buffer (pH 6.0). Endogenous peroxidase activity was inhibited, non-specific binding was blocked. The sections were incubated with primary antibodies for 10 minutes to 12 hours overnight at room temperature. Incubation time and dilution were antibody-specific. Dilutions are listed in Table S1. For example, we diluted 1∶50 for monoclonal antibodies of HIF1A (Thermo Scientific, Frankfurt, Germany) and MUC1 (Zytomed, Berlin, Germany), and 1∶1000 for NOTCH1 (Zytomed). The sections were then incubated with the biotinylated secondary antibody for 10–60 minutes. A final incubation with streptavidin-peroxidase was performed at room temperature for 5–10 minutes. Visualization of the antibodies was achieved with 3,3′-diaminobenzidine or 3-amino-9-ethylcarbazole for 5–10 minutes. Counterstaining was done with Mayeŕs hematoxylin (DAKO, Glostrup, Denmark). Negative controls were performed by leaving out the primary antibody. Staining intensity and proportion of stained epithelial cells in fibrosis-free regions were blindly and independently evaluated by two pathologists (I.S., D.W.). The percentage of cells in intensity groups (none, weak, moderate, or strong) was weighted with factors 0, 1, 2, or 3, respectively, and cumulated in a score for each slide, separately for the membrane, cytoplasm, or nucleus. Supplemental **Figure S1** shows the staining of HIF1A and MUC1 by subtype of lung cancer and **Figure S2** depicts the staining of NOTCH1 in cancer tissue (SqCC) from a miner with high exposure to radon and arsenic.

### Statistical Analysis

The sample size was limited by the availability of tissue blocks with sufficient contrast in exposure to radon and arsenic. Spearman rank correlation coefficients (r_s_) were calculated with 95% confidence intervals (CI) for exploring associations between the staining scores and with exposure or age. Classification methods were applied to the score set to evaluate subtype of lung cancer or level of exposure using R [URL: www.R-project.org], SAS/STAT and SAS/IML software, version 9.2 (SAS Institute Inc., Cary, NC), and TreeView, version 1.60 [18]. Due to skewness, parametric methods were applied to the log-transformed data as ln(score+1). Their correlation structure was further explored with the SAS procedure FACTOR. Hierarchical clustering was performed using Pearson correlation and average linkage in TreeView and with the SAS procedure CLUSTER using Euclidean distance measures and average linkage. Protein profiles were explored with the Classification and Regression Tree Algorithm (CART) as implemented in the rpart library of R (according to Therneau and Atkinson) using a leave-one-out cross-validation.

## Results

### Characteristics of the Study Groups

**Table 1** presents the characteristics of the 146 uranium miners ascertained for the 16 study groups. Age at death ranged from 48 to 87 years. The majority of miners were smokers (95% in lung cancer cases, 88% in cancer-free miners). Silicosis was prevalent in 37% of the miners with and in 64% of the miners without lung cancer and associated with a higher quartz-dust exposure (median 20.9 *vs*. 13.4 mg/m^3^ years, p<0.0001). Cumulative exposure to quartz dust correlated more strongly with exposure to radon (Spearman correlation coefficient r_s_ = 0.78, 95% CI 0.71–0.84) than with exposure to arsenic (r_s_ = 0.31, 95% CI 0.16–0.45).

**Table 1:** Characteristics of 146 uranium miners in 16 strata defined by exposure to radon (Rn) assessed as working level months (WLM) and arsenic (As) and lung cancer.

Exposure	Characteristics	Total	Cancer-free	Adeno-carcinoma	Squamous cell carcinoma	Small cell lung cancer
**Rn high** **As high**(N = 40)	N	40	10	10	10	10
	Age [years] (median, range)	65, 56–84	67, 58–84	62, 56–73	67, 56–77	65, 56–80
	Miners with silicosis	27	9	7	6	5
	Smoking ever/never/missing	33/1/6	8/0/2	9/1/0	8/0/2	8/0/2
	Arsenic [µg/m^3^ years]	229	236	202	327	189
	Radon [WLM]	1879	1987	1763	2041	1368
	Quartz [mg/m^3^ years]	23.9	26.6	24.4	22.6	22.7
**Rn high** **As low**(N = 37)	N	37	10	10	7	10
	Age [years] (median, range)	66, 55–86	70, 58–84	66, 57–81	64, 56–82	62, 55–86
	Miners with silicosis	18	8	5	3	2
	Smoking ever/never/missing	30/2/5	8/1/1	7/1/2	7/0/0	8/0/2
	Arsenic [µg/m^3^ years]	0	0	0	5	9
	Radon [WLM]	1250	1251	1270	1256	1223
	Quartz [mg/m^3^ years]	21.9	20.2	26.8	22.3	19.0
**Rn low** **As high**(N = 33)	N	33	10	7	7	9
	Age [years] (median, range)	66, 48–80	72, 48–79	66, 60–72	70, 55–79	63, 55–80
	Miners with silicosis	14	4	3	2	5
	Smoking ever/never/missing	21/5/7	7/3/0	5/1/1	4/0/3	5/1/3
	Arsenic [µg/m^3^ years]	188	189	188	159	234
	Radon [WLM]	173	63	151	193	182
	Quartz [mg/m^3^ years]	11.7	13.5	9.7	11.2	11.7
**Rn low** **As low**(N = 37)	N	36	9	8	9	10
	Age [years] (median, range)	67, 50–87	75, 50–87	62, 51–73	67, 58–79	71, 52–82
	Miners with silicosis	6	4	0	0	2
	Smoking ever/never/missing	27/0/9	5/0/4	6/0/2	7/0/2	9/0/1
	Arsenic [µg/m^3^ years]	0	0	0	0	0
	Radon [WLM]	54	255	91	58	21
	Quartz [mg/m^3^ years]	5.0	4.8	9.8	5.3	4.9
**Total**	N	146	39	35	33	39
	Age [years] (median, range)	66, 48–87	72, 48–87	63, 51–81	67, 55–82	65, 52–86
	Miners with silicosis	65	25	15	11	14
	Smoking ever/never/missing	111/8/27	28/4/7	27/3/5	26/0/7	30/1/8
	Arsenic [µg/m^3^ years]	75.5	109.2	44.5	104.6	48.0
	Radon [WLM]	1044	1042	1189	1075	1022
	Quartz [mg/m^3^ years]	16.3	17.7	17.3	18.2	13.8

### Associations between Marker Scores

The expression pattern of cancer-related proteins showed strong associations between the 22 markers. These associations remained relatively stable when further stratifying by exposure (supplemental **Table S2**). Three factors were extracted from the correlation matrix that could be attributed to HIF1A, MUC1, and NKX2-1, respectively (data not shown). **Table 2** depicts the Spearman correlation coefficients of the scores of HIF1A, MUC1, and NKX2-1 with the other markers in all tissue samples. In addition, we present the correlations with NOTCH1 as a candidate for exposure-related effects. Similar associations were found if restricted to cancer tissue (data not shown). HIF1A expression was associated with NOTCH1 (r_s_ 0.73, 95% CI 0.65–0.80), ERBB2 (r_s_ 0.58, 95% CI 0.46–0.68) and other proteins except MUC1 and NKX2-1. MUC1 and NKX2-1 were negatively correlated with cancer markers like TP53, VEGFA, or KIT. NOTCH1 and ERBB2 correlated inversely with NKX2-1 (r_s_−0.31, 95% CI−0.45 – −0.15 and −0.30, 95% CI−0.44 – −0.14) but did not show an association with MUC1.

**Table 2:** Spearman correlation coefficients between marker scores in 146 samples of lung tissue.

	HIF1A (cytoplasm)		MUC1 (membrane)		NKX2-1 (nucleus)		NOTCH1 (cytoplasm)	
Marker	r_S_	95% CI	r_S_	95% CI	r_S_	95% CI	r_S_	95% CI
CCND1 (nucleus)	0.69	(0.59;0.77)	−0.07	(−0.23;0.10)	−0.34	(−0.48; −0.19)	0.72	(0.63;0.79)
CCND1 (cytoplasm)	0.73	(0.64;0.80)	−0.27	(−0.42; −0.12)	−0.45	(−0.57; −0.31)	0.78	(0.70;0.83)
CD44 (membrane)	0.58	(0.46;0.68)	−0.08	(−0.24;0.08)	−0.52	(−0.63; −0.39)	0.47	(0.33;0.59)
CD44 (cytoplasm)	0.56	(0.43;0.66)	−0.13	(−0.28;0.04)	−0.45	(−0.57; −0.31)	0.48	(0.34;0.59)
CDH1 (membrane)	0.74	(0.66;0.81)	−0.15	(−0.30;0.01)	−0.43	(−0.55; −0.28)	0.68	(0.58;0.76)
CDH1 (cytoplasm)	0.45	(0.31;0.57)	−0.37	(−0.50; −0.22)	−0.44	(−0.56; −0.29)	0.45	(0.31;0.57)
CTNNB1 (membrane)	0.79	(0.72;0.84)	−0.05	(−0.21;0.11)	−0.40	(−0.53; −0.25)	0.74	(0.65;0.80)
CTNNB1 (cytoplasm)	0.56	(0.44;0.66)	−0.08	(−0.24;0.09)	−0.18	(−0.33; −0.01)	0.53	(0.40;0.64)
EGFR (membrane)	0.69	(0.59;0.77)	−0.08	(−0.24;0.08)	−0.50	(−0.61; −0.37)	0.68	(0.58;0.76)
EGFR (cytoplasm)	0.68	(0.58;0.75)	−0.17	(−0.32;0.00)	−0.46	(−0.58; −0.32)	0.70	(0.61;0.78)
ERBB2 (cytoplasm)	0.58	(0.46;0.68)	−0.10	(−0.25;0.07)	−0.30	(−0.44; −0.14)	0.68	(0.58;0.76)
**HIF1A (cytoplasm)**	1.00		0.15	(−0.02;0.30)	−0.17	(−0.33; −0.01)	0.73	(0.65;0.80)
KIT (cytoplasm)	0.41	(0.27;0.54)	−0.30	(−0.44; −0.14)	−0.25	(−0.40; −0.10)	0.47	(0.33;0.58)
KRT14 (membrane)	0.41	(0.27;0.54)	0.01	(−0.16;0.17)	−0.52	(−0.63; −0.38)	0.38	(0.23;0.51)
KRT14 (cytoplasm)	0.51	(0.38;0.62)	−0.01	(−0.17;0.16)	−0.45	(−0.57; −0.31)	0.47	(0.33;0.59)
KRT5 (membrane)	0.51	(0.38;0.62)	−0.08	(−0.24;0.08)	−0.61	(−0.70; −0.49)	0.47	(0.33;0.59)
KRT5 (cytoplasm)	0.57	(0.45;0.67)	−0.06	(−0.22;0.10)	−0.59	(−0.68; −0.47)	0.54	(0.41;0.64)
MMP2 (cytoplasm)	0.64	(0.53;0.73)	0.16	(0.00;0.31)	−0.07	(−0.23;0.09)	0.58	(0.46;0.68)
**MUC1 (membrane)**	0.15	(−0.02;0.30)	1.00		0.58	(0.46;0.68)	−0.09	(−0.25;0.08)
MUC1 (cytoplasm)	−0.01	(−0.17;0.15)	0.68	(0.57;0.75)	0.70	(0.60;0.77)	−0.13	(−0.28;0.04)
**NKX2-1 (nucleus)**	−0.17	(−0.33; −0.01)	0.58	(0.46;0.68)	1.00		−0.31	(−0.45; −0.15)
**NOTCH1 (cytoplasm)**	0.73	(0.65;0.80)	−0.09	(−0.25;0.08)	−0.31	(−0.45; −0.15)		
PAK1 (nucleus)	0.36	(0.20;0.49)	−0.27	(−0.41; −0.11)	−0.29	(−0.43; −0.14)	0.49	(0.35;0.60)
PTGS2 (cytoplasm)	0.75	(0.66;0.81)	−0.11	(−0.27;0.05)	−0.31	(−0.45; −0.16)	0.71	(0.62;0.78)
SFTPC (cytoplasm)	0.84	(0.78;0.88)	0.35	(0.19;0.48)	−0.04	(−0.20;0.12)	0.68	(0.58;0.76)
SNAI1 (nucleus)	0.43	(0.29;0.55)	−0.41	(−0.54; −0.26)	−0.38	(−0.51; −0.23)	0.45	(0.31;0.57)
SNAI1 (cytoplasm)	0.33	(0.18;0.47)	−0.08	(−0.24;0.08)	−0.16	(−0.32;0.00)	0.40	(0.25;0.52)
STAT3 (nucleus)	0.22	(0.06;0.37)	0.11	(−0.06;0.26)	−0.11	(−0.27;0.05)	0.09	(−0.07;0.25)
STAT3 (cytoplasm)	0.64	(0.53;0.72)	0.17	(0.00;0.32)	−0.02	(−0.18;0.14)	0.55	(0.42;0.65)
TP53 (nucleus)	0.34	(0.18;0.47)	−0.30	(−0.44; −0.14)	−0.34	(−0.48; −0.19)	0.37	(0.22;0.50)
TP53 (cytoplasm)	0.30	(0.14;0.44)	−0.10	(−0.25;0.07)	−0.03	(−0.19;0.13)	0.24	(0.08;0.38)
VEGFA (cytoplasm)	0.55	(0.42;0.65)	−0.38	(−0.51; −0.23)	−0.33	(−0.47; −0.18)	0.55	(0.42;0.65)
VIM (membrane)	0.38	(0.23;0.51)	0.01	(−0.15;0.18)	−0.20	(−0.35; −0.04)	0.31	(0.15;0.45)
VIM (cytoplasm)	0.34	(0.18;0.47)	0.02	(−0.14;0.19)	−0.08	(−0.24;0.09)	0.36	(0.21;0.49)

### Protein Expression by Exposure to Radon and Arsenic

Supplemental **Table S3** depicts the distribution of positively stained samples by level of exposure to radon and arsenic. In lung tissue from uranium miners with high exposure to both carcinogens, up-regulation of cancer-related proteins was more common than in miners with low exposure. Seven out of 18 cytoplasmic proteins were more frequently stained (≥15%) in the high-exposed tissue samples, among these ERBB2 and NOTCH1. None of the samples from low-exposed miners had a similar fraction (≥15%) of stained samples in excess to the high-exposure group.

**Table 3** (selected proteins) and supplemental **Table S4** (all proteins) show the correlation of the staining scores with cumulative exposure to radon (assessed as WLM) and arsenic in all samples and stratified by lung cancer. Up-regulation of cancer-related proteins was frequently observed with increasing exposure to radon in lung-cancer tissue, but we detected no significant down-regulation. Radon exposure correlated further with TP53 in cancer-free tissue (r_s_ 0.40, 95% CI 0.09–0.63). The effects of exposure to arsenic on the staining scores were less clear. ERBB2 and NOTCH1 showed an up-regulation in lung-cancer tissue with increased exposure to both carcinogens, but no staining or a lacking association with exposure in cancer-free samples.

**Table 3:** Spearman correlation coefficients of selected marker scores with cumulative exposure to radon and arsenic in lung tissue from 146 uranium miners.

	Exposure	NOTCH1 (cytoplasm)		HIF1A (cytoplasm)		MUC1 (membrane)		NKX2-1 (nucleus)	
		r_S_	95% CI	r_S_	95% CI	r_S_	95% CI	r_S_	95% CI
All (N = 146)	Radon	0.18	(0.02;0.33)	0.12	(−0.04;0.28)	0.03	(−0.13;0.19)	0.07	(−0.10;0.23)
	Arsenic	0.23	(0.07;0.38)	0.03	(−0.13;0.19)	0.00	(−0.16;0.16)	0.01	(−0.15;0.17)
Lung cancer (N = 107)	Radon	0.26	(0.07;0.42)	0.12	(−0.07;0.30)	0.07	(−0.12;0.26)	0.11	(−0.08;0.29)
	Arsenic	0.34	(0.16;0.49)	0.02	(−0.17;0.21)	0.06	(−0.14;0.24)	0.00	(−0.19;0.19)
Adenocarcinoma (N = 35)	Radon	0.31	(−0.03;0.58)	0.13	(−0.21;0.44)	0.16	(−0.18;0.47)	−0.07	(−0.39;0.27)
	Arsenic	0.73	(0.51;0.85)	0.05	(−0.29;0.37)	0.11	(−0.23;0.43)	−0.04	(−0.36;0.30)
Squamous cell carcinoma (N = 33)	Radon	0.34	(−0.01;0.61)	−0.06	(−0.40;0.29)	0.10	(−0.26;0.42)	–	
	Arsenic	0.25	(−0.11;0.54)	−0.05	(−0.39;0.30)	−0.03	(−0.37;0.32)	–	
Small cell lung cancer (N = 39)	Radon	0.22	(−0.10;0.50)	0.21	(−0.11;0.49)	−0.20	(−0.48;0.13)	0.46	(0.16;0.67)
	Arsenic	0.28	(−0.05;0.54)	0.00	(−0.31;0.32)	0.16	(−0.16;0.45)	0.14	(−0.19;0.43)
Cancer-free samples (N = 39)	Radon	−0.12	(−0.42;0.20)	0.10	(−0.22;0.40)	0.12	(−0.20;0.42)	0.17	(−0.16;0.46)
	Arsenic	−0.12	(−0.42;0.20)	0.03	(−0.29;0.34)	−0.32	(−0.57;0.00)	0.17	(−0.16;0.46)

### Staining Results by Subtype of Lung Cancer

**Table 4** shows the distribution of positively stained samples by major subtype of lung cancer and in cancer-free tissue. Most slides from cancer-free tissue lacked expression of CCND1, CD44, CDH1, EGFR, ERBB2, KIT, keratins, NOTCH1, PAK1, PTGS2, SNAI1, and VIM but showed staining of MUC1, HIF1A, NKX2-1, SFTPC, and STAT3, which were also found in AdCa. Membrane staining of MUC1, SFTPC in cytoplasm, and many other markers were frequently lacking in SCLC. AdCa and SqCC shared signatures, including ERBB2, KIT, MMP2, PTGS2, EGFR, and VEGFA. KRT5 and KRT14 were more frequently expressed in SqCC than in AdCa, whereas NKX2-1 was lacking.

**Table 4:** Proportion of lung tissue samples with positive staining of candidate proteins in lung cancer tissue and in cancer-free samples from uranium miners.

	Adenocarcinoma (N = 35)			Squamous cell carcinoma (N = 33)			Small cell lung cancer (N = 39)			Cancer-free (N = 39)		
	Membrane	Cytoplasm	Nucleus	Membrane	Cytoplasm	Nucleus	Membrane	Cytoplasm	Nucleus	Membrane	Cytoplasm	Nucleus
**CCND1**	0	33 (94%)	28 (80%)	4 (12%)	33 (100%)	27 (82%)	0	31 (79%)	1 (3%)	0	2 (5%)	0
**CD44**	15 (43%)	17 (49%)	0	29 (88%)	28 (85%)	0	2 (5%)	4 (10%)	0	0	0	0
**CDH1**	32 (91%)	28 (80%)	0	33 (100%)	33 (100%)	0	15 (38%)	21 (54%)	0	0	0	0
**CTNNB1**	31 (89%)	29 (83%)	1 (3%)	32 (97%)	30 (91%)	0	6 (15%)	19 (49%)	0	0	18 (46%)	0
**EGFR**	27 (77%)	31 (89%)	0	33 (100%)	33 (100%)	0	7 (18%)	14 (36%)	0	0	0	0
**ERBB2**	1 (3%)	23 (66%)	0	0	25 (76%)	0	0	10 (26%)	0	0	0	0
**HIF1A**	0	35 (100%)	0	0	33 (100%)	0	0	22 (56%)	0	0	35 (90%)	3 (8%)
**KIT**	3 (9%)	24 (69%)	0	0	23 (70%)	0	0	21 (54%)	0	0	0	0
**KRT5**	5 (14%)	9 (26%)	0	32 (97%)	33 (100%)	0	1 (3%)	1 (3%)	0	0	0	0
**KRT14**	1 (3%)	8 (23%)	0	22 (67%)	23 (70%)	0	1 (3%)	3 (8%)	0	0	0	0
**MMP2**	0	27 (77%)	0	0	24 (73%)	0	0	2 (5%)	0	0	16 (41%)	0
**MUC1**	29 (83%)	34 (97%)	0	32 (97%)	33 (100%)	0	4 (10%)	37 (95%)	0	39 (100%)	39 (100%)	0
**NKX2-1**	0	0	35 (100%)	0	0	0	0	0	29 (74%)	0	0	39 (100%)
**NOTCH1**	0	29 (83%)	1 (3%)	0	31 (94%)	3 (9%)	0	10 (26%)	0	0	4 (10%)	0
**PAK1**	0	7 (20%)	23 (66%)	0	2 (6%)	22 (67%)	0	1 (3%)	17 (44%)	0	0	0
**PTGS2**	1 (3%)	31 (89%)	0	0	30 (91%)	1 (3%)	0	10 (26%)	0	0	0	0
**SFTPC**	0	33 (94%)	1 (3%)	0	33 (100%)	0	0	7 (18%)	0	0	38 (97%)	0
**SNAI1**	0	8 (23%)	35 (100%)	0	9 (27%)	30 (91%)	0	3 (8%)	28 (72%)	0	0	2 (5%)
**STAT3**	0	29 (83%)	6 (17%)	0	32 (97%)	14 (42%)	0	25 (64%)	3 (8%)	0	39 (100%)	10 (26%)
**TP53**	0	9 (26%)	34 (97%)	0	6 (18%)	32 (97%)	0	5 (13%)	34 (87%)	0	0	27 (69%)
**VEGFA**	0	34 (97%)	2 (6%)	0	33 (100%)	3 (9%)	0	29 (74%)	2 (5%)	2 (5%)	6 (15%)	0
**VIM**	6 (17%)	12 (34%)	0	8 (24%)	11 (33%)	0	0	5 (13%)	0	0	3 (8%)	0

### Classification of Protein Patterns by Subtype of Lung Cancer

**Figure 1:**
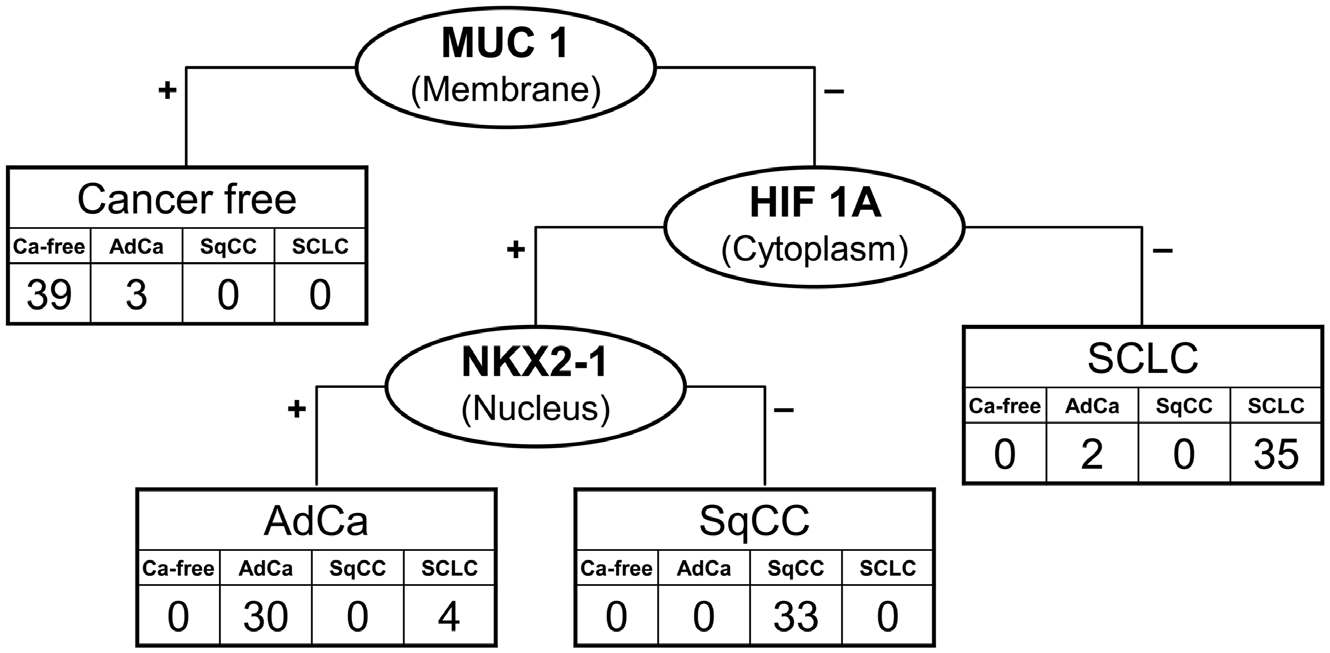
Classification and regression tree with leave-one-out cross-validation for cancer-free lung tissue, adenocarcinoma (AdCa), squamous cell carcinoma (SqCC), and small cell cancer of the lung (SCLC). using immunohistochemical scores of 22 candidate proteins in 146 tissue samples from uranium miners. Staining of mucin 1 (MUC1) in the membrane, hypoxia-inducible factor 1 α (HIF1A) in the cytoplasm, and NK2 homeobox 1 (NKX2-1) in the nucleus resulted in a three-protein classifier with a failure rate of 11.0%.

**Figure 2:**
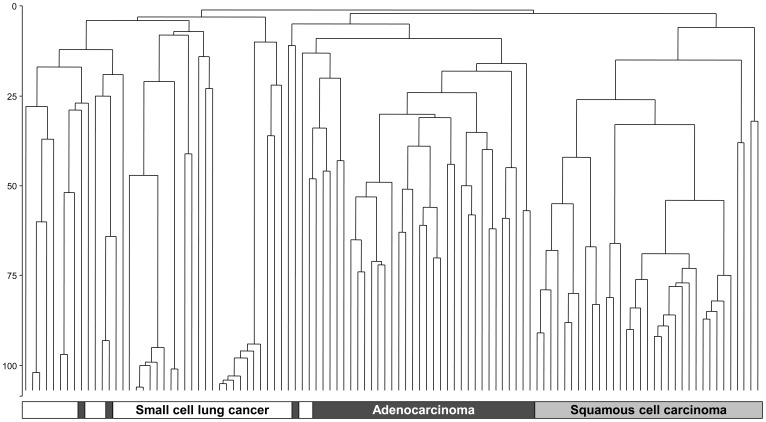
Cluster analysis of major subtypes of lung cancer in 107 tissue samples from uranium miners using average linkage and Euclidian distance for immunohistochemical scores of hypoxia-inducible factor 1 α (HIF1A) in the cytoplasm and NKX2-1 in the nucleus.

**Figure 1** depicts the CART classification of lung cancer in all tissue samples and **Figure 2** shows the clustering of cancer-tissue samples by major histological subtype. Staining of MUC1 (membrane), HIF1A (cytoplasm), and NKX2-1 (nucleus) classified cancer by subtype and cancer-free lung tissue with a failure rate of 11.0%. Higher MUC1 expression in the membrane discriminated normal tissue from cancer (failure rate 2.1%). HIF1A was expressed in NSCLC but less in SCLC, whereas NKX2-1 was lacking in SqCC. HIF1A and NKX2-1 classified the major subtypes of lung cancer with a failure rate of 8.4%. EGFR and VEGFA classified NSCLC *vs*. SCLC with a failure rate of 10.3% and AdCa *vs*. SqCC with 19.1%. We confirmed the 2-protein signature for the discrimination of the major subtypes of lung cancer with 15 additional samples retrieved from the archive. All five SCLC cases showed a very weak or lacking staining of HIF1A, and all five AdCa cases were presented with high NKX2-1 scores that were zero in SqCC or low in SCLC.

Exposure to radon or arsenic could not be clearly detected in the 22-protein signatures, with a failure rate of 62.6% in cancer tissue and 57.5% in all samples (data not shown). Failure rates between 37% and 39% were found for smoking and silicosis, respectively. It is important to note that most miners were smokers, and staining was investigated in fibrosis-free parts of the tissue samples.

## Discussion

Lung cancer was a common occupational disease in German uranium miners with an excess risk of radon exposure [4]. Increasing exposure to radon or arsenic was associated with a shift towards SCLC or SqCC at the expense of AdCa [11], [12]. This raised the question whether exposure-related changes can be detected in expression patterns in the lungs of miners. We observed an up-regulation of cancer-related proteins, including NOTCH1, with increasing cumulative exposure to radon or arsenic, but could not detect an additional effect of exposure on the very distinct patterns of proteins of the major subtypes of lung cancer. A tight correlation structure between the staining scores revealed three proteins that served as good diagnostic classifiers. MUC1 discriminated cancer-free tissue from lung cancer. HIF1A and NKX2-1 discriminated the major subtypes with a low failure rate.

The correlation of NOTCH1 expression with exposure to radon is in line with experimental results. *NOTCH1*, a large gene comprising 37 exons, is prone to radiation-induced mutations that can contribute to T-cell lymphomagenesis [9], [19]. Up-regulation of NOTCH was also observed after irradiation of embryonic kidney cells [20], and down-regulation rendered glioma stem cells more sensitive to radiation [21]. Less is known about NOTCH1 in lung cancer cases with radiation exposure. In our study, NOTCH1 was constitutively active in most NSCLC samples but less in SCLC and lacking in cancer-free tissue.

NOTCH1 was also upregulated in lung cancer samples from uranium miners with high exposure to arsenic. This may be partially due to a moderate correlation between arsenic and radon. Keratins have emerged as a relevant target of arsenic [22]. Whereas cytokeratins contributed to the specific staining of SqCC they did not show an additional preference for arsenic exposure.

NOTCH1 correlated with the expression of various other cancer-related proteins like HIF1A but not with MUC1 as a marker of normal lung physiology. Mucus production is a primary particle-defense mechanism of the airways [23]. Downregulation of membrane-bound MUC1 discriminated cancer-free tissue from cancer tissue. This supports the view that changes in mucin biosynthesis can serve as a tumor marker [24].

Treatment decision of lung cancer usually rests on the categorization into NSCLC and SCLC. In our study, the commonly applied markers VEGFA and EGFR separated both lineages but were of limited value to further discriminate between AdCa and SqCC. Both entities have shown different responses to therapy [25]. Our results revealed HIF1A together with NKX2-1 as a two-protein panel that discriminated all major subtypes with a low failure rate. HIF1A expression was lower in SCLC, and NKX2-1 staining was lacking in SqCC. We could confirm that the protein patterns of SqCC and SCLC indicate a greater ‘stemness’ whereas the molecular signatures of AdCa represent more differentiated stages [26], [27]. AdCa is a peripheral tumor and continues expressing proteins typical of the lung physiology such as mucins, surfactant proteins, or NKX2-1.

Hypoxia is a basic feature of cancer where HIF1A has been identified as a key regulator of energy metabolism and other oncogenic pathways [28], [29]. Many target genes have been identified, including *VEGF*, *VIM*, and *KRT14* [30]. In our study, HIF1A, similarly to NOTCH1, correlated with most of the other tumor markers but less with MUC1 or NKX2-1 as markers of lung physiology. A higher score of cytoplasmic staining classified NSCLC from SCLC samples. *HIF1A* mRNA has been observed to be up-regulated in NSCLC [31] and suggested as a prognostic classifier [32], [33]. HIF1A might also constitute a therapeutic target [34], [35].

*NKX2-1* has been found frequently amplified and overexpressed in AdCa [36] and is an established marker of lung-cancer lineage used to distinguish AdCa from the more centrally located SqCC. We confirmed its expression in AdCa whereas staining was lacking in SqCC. *NKX2-1* is essential for the formation of alveolar type 2 (AT2) pneumocytes [37]. Both AT2 cells and AdCa are located in distant parts of the lung, where mucins keep the epithelial layer hydrated and act together with surfactants as a filtration barrier [38].

Various methodological shortcomings have to be taken into account when studying lung cancer. The classification of subtypes is prone to observer bias [39]. Here, lung tissue was available from autopsies and subject to reference pathology. Another issue concerns misclassification of exposure [40]. Enormous efforts have been undertaken to assess occupational exposure to radon and arsenic in uranium mining [2], [3]. Exposure to radon and arsenic can result in a synergistic action. Accordingly, more samples were positively stained in the group with high exposure to both carcinogens than in the low-exposed group.

In this particular context of heavy occupational exposure, confounding by smoking was estimated to be of minor concern [5]. There was no strong variation of smoking prevalence by level of exposure. No obvious effect of smoking was found in miRNA patterns in a large set of AdCa samples, where also a good molecular classification of AdCa and SqCC could be achieved [41].

Similarly, our markers were also good classifiers of the subtypes, but we could not identify an additional effect of exposure on the subtype-specific patterns. Although we were able to detect a moderate association between exposure and NOTCH1 and other proteins, the strong differences in expression by subtype might hinder the detection of weaker influences. This raised the question whether our study design was powerful enough to detect such modification in expression levels. A first investigation with cDNA microarrays in thyroid tumors, including samples from the Chernobyl Tissue Bank, revealed no radiation-specific signature [42]. A subsequent analysis allowed the identification of a subtle gene expression signature in a subgroup of Chernobyl cases, which were susceptible to radiation-induced cancer [8]. We had chosen an orthogonal study design with contrast in exposure. Although the tissue bank is rather comprehensive, the tissue blocks were limited for rare combinations like low radon and high arsenic. Furthermore, an extensive stratification results in smaller subgroups that are prone to variation by chance. We therefore paid attention to consistent trends in the results.

Another concern is the question if the methodology used was suitable and the resulting protein set was sufficiently complete and sensitive. Modern mass spectrometry-based proteomics has made great progress in its application to archival material [43], [44]. Instead of using a method for global protein analysis, however, we have chosen a hypothesis-driven approach that was based on immunohistochemistry. Antigen retrieval with archival material is well established and, despite long fixation times in unbuffered formalin, 22 out of 30 antibodies could be successfully applied. Initially, we searched the literature for candidate proteins being employed in lung cancer, development, or physiology. The observed strong associations between the scores of the 22 proteins support the candidate protein approach. The tight correlation structure could be represented by HIF1A, MUC1, and NKX2-1. This dimensionality reduction might be due to the contribution of various key proteins to fundamental pathways. Although we cannot exclude missing important proteins in the set chosen for this investigation, for example from DNA repair pathways, the correlation structure of the candidate protein supports the view that such an analysis can similarly be conducted with proxy proteins. If exposure influenced a candidate protein, such an effect could be transported along the pathways or across networks of key proteins.

Apart from methodological limitations, biological explanations should also be discussed as to why exposure might cause a shift between the subtypes. Many of the markers are employed in developmental pathways of lung morphogenesis that are recapitulated in tissue regeneration and cancer [45]–[47]. These programs result in lineage-specific expression patterns that can be well classified by these proteins. It is current opinion that at least two major lineages give rise to SCLC and NSCLC [48], [49]. SCLC is a more aggressive tumor with neuroendocrine features [50]. AdCa is the common subtype in never smokers and more differentiated than SqCC within the NSCLC lineage [26], [27]. Repair of heavy tissue damage usually needs reconstruction of a more complex tissue architecture and implies recruitment of cells with higher stemness [51]. This may explain the observed shift in the distribution of the subtypes of lung cancer by level of exposure to carcinogens. Exposure to radon or arsenic may cause the transformation of a stem cell to a cancer stem cell. However, tightly controlled programs activated in the process of carcinogenesis might obscure the transfer of exposure-related damage in a precursor cell along the lineage to a specific cancer phenotype.

In conclusion, NOTCH1, a prominent candidate for radiation-related effects, and other cancer-related proteins were associated weakly to moderately with exposure to radon and arsenic. MUC1, a physiological marker, HIF1A, a regulator of metabolic reprogramming, and NKX2-1, a lineage-specific marker performed well as classifiers of lung cancer and its major subtypes.

## Supporting Information

Figure S1 **Staining of mucin 1 (MUC1) (right column) in the membrane and hypoxia-inducible factor 1 α (HIF1A) (left column) in the cytoplasm of archived lung tissue from uranium miners, showing cancer-free lung tissue, adenocarcinoma (AdCa), squamous cell carcinoma (SqCC), and small cell cancer of the lung (SCLC) (descending).**
(JPG)

Figure S2 **Staining of NOTCH1 in a sample of squamous cell carcinoma from one miner with high exposure to radon and arsenic.**
(TIF)

Table S1 **Candidate proteins selected for immunostaining of archived lung tissue.**
(DOC)

Table S2 **Spearman correlation coefficients between marker scores in lung tissue from uranium miners by level of exposure to radon and arsenic.**
(DOC)

Table S3 **Proportion of samples with positive staining of candidate proteins in lung cancer and in cancer-free tissue from uranium miners by level of exposure to radon and arsenic.**
(DOC)

Table S4 **Spearman correlation coefficients of marker scores with cumulative exposure to radon [WLM] and arsenic [µg/m^3^ years] in lung tissue from uranium miners.**
(DOC)
