# Supplementary figures and images for: NOTCH1, HIF1A and Other Cancer-Related Proteins in Lung Tissue from Uranium Miners—Variation by Occupational Exposure and Subtype of Lung Cancer
Source: PLoS One. 2012 Sep 17;7(9):e45305. doi: 10.1371/journal.pone.0045305 (PMC3444449; doi:10.1371/journal.pone.0045305)

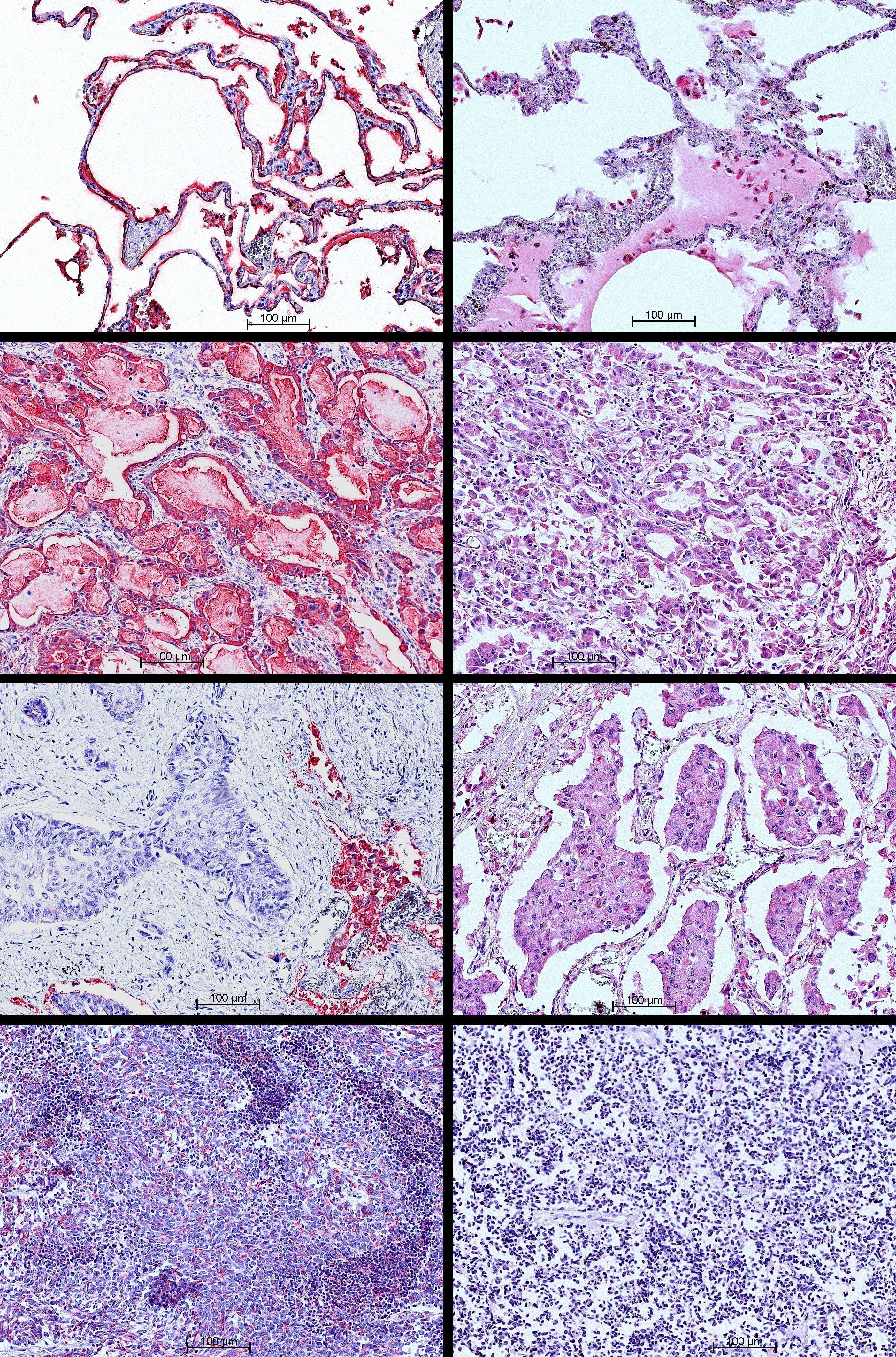

Supplement: Figure S1 — Staining of mucin 1 (MUC1) (right column) in the membrane and hypoxia-inducible factor 1 α (HIF1A) (left column) in the cytoplasm of archived lung tissue from uranium miners, showing cancer-free lung tissue, adenocarcinoma (AdCa), squamous cell carcinoma (SqCC), and small cell cancer of the lung (SCLC) (descending). (JPG) [file pone.0045305.s001.jpg]

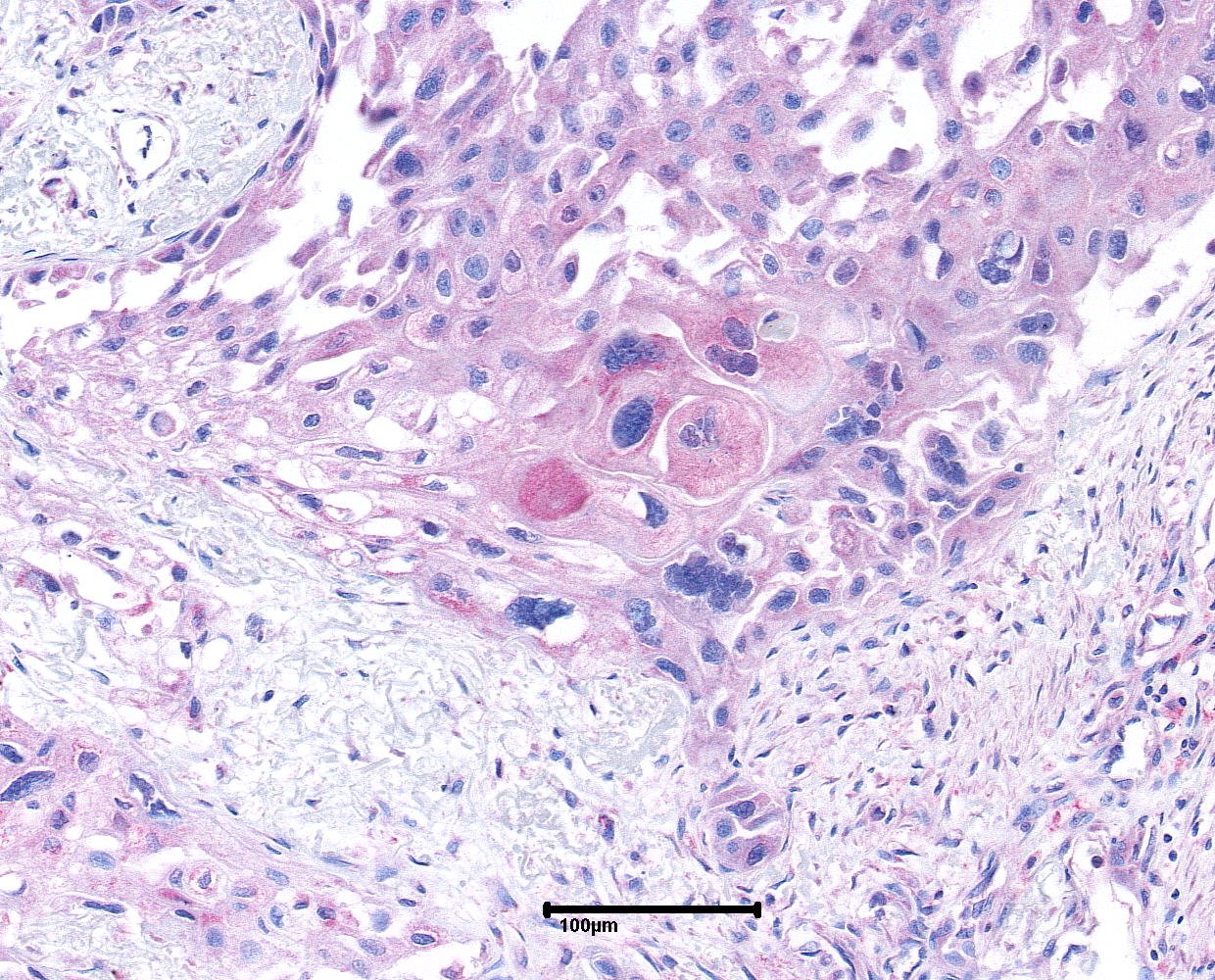

Supplement: Figure S2 — Staining of NOTCH1 in a sample of squamous cell carcinoma from one miner with high exposure to radon and arsenic. (TIF) [file pone.0045305.s002.tif]
