# Supplementary material for: NOTCH1, HIF1A and Other Cancer-Related Proteins in Lung Tissue from Uranium Miners—Variation by Occupational Exposure and Subtype of Lung Cancer
Source: PLoS One. 2012 Sep 17;7(9):e45305. doi: 10.1371/journal.pone.0045305 (PMC3444449; doi:10.1371/journal.pone.0045305)
Supplement: Table S1 — Candidate proteins selected for immunostaining of archived lung tissue. (DOC) [file pone.0045305.s003.doc]

**Table S1. Candidate proteins selected for immunostaining of archived lung tissue**

| **Symbol** | **Name** | **Dilution** | **Antibody** |
| --- | --- | --- | --- |
| CCND1 | Cyclin D1 | 1:50 | monoclonal |
| CD44 | CD44 molecule (Indian blood group) | 1:100 | monoclonal |
| CDH1 | Cadherin 1, type1, E-cadherin (epithelial) | 1:100 | monoclonal |
| KIT | v-kit Hardy-Zuckerman 4 feline sarcoma viral oncogene homolog | 1:200 | polyclonal |
| PTGS2 (COX2) | Prostaglandin-endoperoxide synthase 2 | 1:50 | monoclonal |
| CTNNB1 | Catenin (cadherin-associated protein), beta 1 | 1:300 | polyclonal |
| EGFR | Epidermal growth factor receptor | 1:50 | monoclonal |
| ERBB2 | v-erb-b2 erythroblastic leukemia viral oncogene homolog 2 | 1:600 | polyclonal |
| HIF1A | Hypoxia-inducible factor 1, alpha subunit  (basic helix-loop-helix transcription factor) | 1:50 | monoclonal |
| KRT5 | Keratin 5 | 1:200 | monoclonal |
| KRT14 | Keratin 14 | 1:50 | monoclonal |
| MMP2 | Matrix metallopeptidase 2 | 1:50 | monoclonal |
| MUC 1 | Mucin 1 | 1:50 | monoclonal |
| NKX2-1 (TTF1) | NK2 homeobox 1 | 1:50 | monoclonal |
| NOTCH1 | Neurogenic locus notch homolog protein 1 | 1:1000 | polyclonal |
| TP53 | Tumor protein p53 | 1:20 | monoclonal |
| PAK1 (P21) | P21 protein, (CDC42/Rac)-activated kinase 1 | 1:50 | monoclonal |
| SNAI1 | Snail homolog 1 | 1:75 | polyclonal |
| SFTPC | Surfactant protein C | 1:200 | polyclonal |
| STAT3 | Signal transducer and activator of transcription 3 | 1:500 | polyclonal |
| VEGFA | Vascular endothelial growth factor A | 1:20 | monoclonal |
| VIM | Vimentin | 1:12000 | monoclonal |
