# Supplementary material for: NOTCH1, HIF1A and Other Cancer-Related Proteins in Lung Tissue from Uranium Miners—Variation by Occupational Exposure and Subtype of Lung Cancer
Source: PLoS One. 2012 Sep 17;7(9):e45305. doi: 10.1371/journal.pone.0045305 (PMC3444449; doi:10.1371/journal.pone.0045305)
Supplement: Table S2 — Spearman correlation coefficients between marker scores in lung tissue from uranium miners by level of exposure to radon and arsenic. (DOC) [file pone.0045305.s004.doc]

**Table S2. Spearman correlation coefficients between marker scores in lung tissue from uranium miners by level of exposure to radon and arsenic**

|  | **High radon and high arsenic exposure (N=40)** | | | | | | | |
| --- | --- | --- | --- | --- | --- | --- | --- | --- |
|  | **HIF1A (cytoplasm)** | | **MUC1 (membrane)** | | **NKX2-1 (nucleus)** | | **NOTCH1 (cytoplasm)** | |
| **Marker** | **r_S_** | **95% CI** | **r_S_** | **95% CI** | **r_S_** | **95% CI** | **r_S_** | **95% CI** |
| CCND1 (nucleus) | 0.68 | (0.46; 0.82) | 0.04 | (-0.28; 0.34) | -0.45 | (-0.66; -0.16) | 0.77 | (0.60; 0.87) |
| CCND1 (cytoplasm) | 0.76 | (0.59; 0.87) | -0.25 | (-0.52; 0.07) | -0.41 | (-0.63; -0.10) | 0.89 | (0.80; 0.94) |
| CD44 (membrane) | 0.42 | (0.12; 0.64) | -0.06 | (-0.37; 0.25) | -0.60 | (-0.76; -0.34) | 0.42 | (0.12; 0.64) |
| CD44 (cytoplasm) | 0.52 | (0.25; 0.72) | -0.06 | (-0.37; 0.25) | -0.41 | (-0.63; -0.10) | 0.55 | (0.28; 0.73) |
| CDH1 (membrane) | 0.72 | (0.51; 0.84) | -0.14 | (-0.43; 0.18) | -0.52 | (-0.71; -0.24) | 0.80 | (0.65; 0.89) |
| CDH1 (cytoplasm) | 0.26 | (-0.06; 0.53) | -0.52 | (-0.71; -0.24) | -0.53 | (-0.72; -0.26) | 0.43 | (0.13; 0.65) |
| CTNNB1 (membrane) | 0.78 | (0.62; 0.88) | -0.03 | (-0.33; 0.29) | -0.36 | (-0.60; -0.04) | 0.86 | (0.74; 0.92) |
| CTNNB1 (cytoplasm) | 0.38 | (0.08; 0.62) | -0.20 | (-0.48; 0.12) | -0.11 | (-0.41; 0.21) | 0.45 | (0.15; 0.66) |
| EGFR (membrane) | 0.56 | (0.30; 0.74) | 0.01 | (-0.30; 0.32) | -0.55 | (-0.73; -0.28) | 0.67 | (0.45; 0.81) |
| EGFR (cytoplasm) | 0.65 | (0.42; 0.80) | -0.01 | (-0.32; 0.30) | -0.46 | (-0.67; -0.17) | 0.77 | (0.59; 0.87) |
| ERBB2 (cytoplasm) | 0.56 | (0.30; 0.74) | -0.12 | (-0.41; 0.20) | -0.49 | (-0.69; -0.21) | 0.74 | (0.55; 0.85) |
| HIF1A (cytoplasm) | 1.00 |  | 0.21 | (-0.11; 0.49) | -0.19 | (-0.47; 0.13) | 0.82 | (0.68; 0.90) |
| KIT (cytoplasm) | 0.45 | (0.16; 0.67) | -0.23 | (-0.50; 0.09) | -0.31 | (-0.57; 0.00) | 0.57 | (0.31; 0.74) |
| KRT14 (membrane) | 0.38 | (0.07; 0.62) | 0.05 | (-0.26; 0.36) | -0.42 | (-0.65; -0.12) | 0.40 | (0.09; 0.63) |
| KRT14 (cytoplasm) | 0.52 | (0.24; 0.71) | 0.09 | (-0.23; 0.39) | -0.20 | (-0.48; 0.12) | 0.57 | (0.31; 0.75) |
| KRT5 (membrane) | 0.39 | (0.08; 0.62) | -0.03 | (-0.34; 0.29) | -0.72 | (-0.84; -0.52) | 0.44 | (0.15; 0.66) |
| KRT5 (cytoplasm) | 0.49 | (0.21; 0.70) | -0.03 | (-0.34; 0.28) | -0.63 | (-0.78; -0.39) | 0.56 | (0.30; 0.74) |
| MMP2 (cytoplasm) | 0.65 | (0.41; 0.79) | 0.19 | (-0.13; 0.47) | 0.02 | (-0.29; 0.33) | 0.68 | (0.46; 0.81) |
| MUC1 (membrane) | 0.21 | (-0.11; 0.49) | 1.00 |  | 0.50 | (0.21; 0.70) | 0.01 | (-0.30; 0.32) |
| MUC1 (cytoplasm) | 0.11 | (-0.21; 0.40) | 0.65 | (0.42; 0.80) | 0.71 | (0.51; 0.84) | 0.03 | (-0.29; 0.34) |
| NKX2-1 (nucleus) | -0.19 | (-0.47; 0.13) | 0.50 | (0.21; 0.70) | 1.00 |  | -0.34 | (-0.59; -0.03) |
| NOTCH1 (cytoplasm) | 0.82 | (0.68; 0.90) | 0.01 | (-0.30; 0.32) | -0.34 | (-0.59; -0.03) | 1.00 |  |
| PAK1 (nucleus) | 0.43 | (0.13; 0.65) | -0.26 | (-0.53; 0.06) | -0.37 | (-0.61; -0.06) | 0.56 | (0.29; 0.74) |
| PTGS2 (cytoplasm) | 0.68 | (0.45; 0.81) | -0.07 | (-0.37; 0.25) | -0.32 | (-0.57; 0.00) | 0.84 | (0.72; 0.91) |
| SFTPC (cytoplasm) | 0.88 | (0.79; 0.94) | 0.29 | (-0.02; 0.55) | -0.07 | (-0.37; 0.25) | 0.86 | (0.75; 0.92) |
| SNAI1 (nucleus) | 0.51 | (0.22; 0.70) | -0.35 | (-0.60; -0.04) | -0.32 | (-0.57; -0.01) | 0.62 | (0.38; 0.78) |
| SNAI1 (cytoplasm) | 0.47 | (0.17; 0.68) | -0.12 | (-0.41; 0.20) | -0.33 | (-0.58; -0.02) | 0.43 | (0.13; 0.65) |
| STAT3 (nucleus) | 0.07 | (-0.25; 0.37) | 0.01 | (-0.30; 0.32) | -0.09 | (-0.39; 0.23) | -0.03 | (-0.34; 0.28) |
| STAT3 (cytoplasm) | 0.62 | (0.38; 0.78) | -0.04 | (-0.35; 0.27) | -0.05 | (-0.36; 0.27) | 0.68 | (0.46; 0.82) |
| TP53 (nucleus) | 0.25 | (-0.07; 0.52) | -0.27 | (-0.53; 0.05) | -0.20 | (-0.48; 0.12) | 0.48 | (0.19; 0.69) |
| TP53 (cytoplasm) | 0.18 | (-0.14; 0.47) | -0.02 | (-0.33; 0.29) | -0.14 | (-0.43; 0.18) | 0.13 | (-0.19; 0.42) |
| VEGFA (cytoplasm) | 0.41 | (0.11; 0.64) | -0.42 | (-0.64; -0.12) | -0.26 | (-0.53; 0.06) | 0.50 | (0.22; 0.70) |
| VIM (membrane) | 0.33 | (0.02; 0.58) | 0.04 | (-0.27; 0.35) | -0.13 | (-0.42; 0.19) | 0.44 | (0.14; 0.66) |
| VIM (cytoplasm) | 0.24 | (-0.08; 0.51) | -0.19 | (-0.47; 0.14) | -0.09 | (-0.39; 0.23) | 0.49 | (0.20; 0.69) |

**Table S2. Spearman correlation coefficients between marker scores in lung tissue from uranium miners by level of exposure to radon and arsenic (continued)**

|  | **High radon and low arsenic exposure (N=37)** | | | | | | | |
| --- | --- | --- | --- | --- | --- | --- | --- | --- |
|  | **HIF1A (cytoplasm)** | | **MUC1 (membrane)** | | **NKX2-1 (nucleus)** | | **NOTCH1 (cytoplasm)** | |
| **Marker** | **r_S_** | **95% CI** | **r_S_** | **95% CI** | **r_S_** | **95% CI** | **r_S_** | **95% CI** |
| CCND1 (nucleus) | 0.71 | (0.49; 0.84) | -0.08 | (-0.39; 0.25) | -0.41 | (-0.64; -0.09) | 0.64 | (0.40; 0.80) |
| CCND1 (cytoplasm) | 0.79 | (0.62; 0.89) | -0.26 | (-0.54; 0.07) | -0.68 | (-0.82; -0.45) | 0.67 | (0.43; 0.81) |
| CD44 (membrane) | 0.64 | (0.39; 0.80) | -0.12 | (-0.43; 0.21) | -0.54 | (-0.73; -0.25) | 0.49 | (0.20; 0.70) |
| CD44 (cytoplasm) | 0.60 | (0.33; 0.77) | -0.14 | (-0.44; 0.20) | -0.41 | (-0.64; -0.09) | 0.44 | (0.13; 0.67) |
| CDH1 (membrane) | 0.73 | (0.53; 0.85) | -0.26 | (-0.54; 0.07) | -0.52 | (-0.72; -0.23) | 0.60 | (0.34; 0.77) |
| CDH1 (cytoplasm) | 0.43 | (0.12; 0.66) | -0.51 | (-0.71; -0.22) | -0.53 | (-0.73; -0.25) | 0.45 | (0.14; 0.67) |
| CTNNB1 (membrane) | 0.81 | (0.66; 0.90) | -0.09 | (-0.40; 0.24) | -0.52 | (-0.72; -0.23) | 0.65 | (0.40; 0.80) |
| CTNNB1 (cytoplasm) | 0.37 | (0.05; 0.62) | -0.21 | (-0.50; 0.12) | -0.38 | (-0.62; -0.06) | 0.32 | (-0.01; 0.58) |
| EGFR (membrane) | 0.67 | (0.43; 0.81) | -0.12 | (-0.43; 0.21) | -0.62 | (-0.78; -0.36) | 0.56 | (0.28; 0.75) |
| EGFR (cytoplasm) | 0.66 | (0.42; 0.81) | -0.24 | (-0.52; 0.10) | -0.60 | (-0.77; -0.34) | 0.49 | (0.20; 0.70) |
| ERBB2 (cytoplasm) | 0.48 | (0.18; 0.69) | -0.14 | (-0.44; 0.19) | -0.24 | (-0.52; 0.09) | 0.62 | (0.37; 0.78) |
| HIF1A (cytoplasm) | 1.00 |  | 0.10 | (-0.23; 0.41) | -0.36 | (-0.61; -0.04) | 0.68 | (0.45; 0.82) |
| KIT (cytoplasm) | 0.33 | (0.00; 0.58) | -0.46 | (-0.68; -0.15) | -0.36 | (-0.61; -0.04) | 0.57 | (0.29; 0.75) |
| KRT14 (membrane) | 0.31 | (-0.02; 0.57) | 0.09 | (-0.24; 0.40) | -0.40 | (-0.64; -0.08) | 0.54 | (0.25; 0.73) |
| KRT14 (cytoplasm) | 0.47 | (0.16; 0.68) | -0.07 | (-0.38; 0.26) | -0.51 | (-0.71; -0.22) | 0.55 | (0.26; 0.74) |
| KRT5 (membrane) | 0.58 | (0.30; 0.76) | -0.11 | (-0.42; 0.22) | -0.59 | (-0.76; -0.32) | 0.56 | (0.28; 0.75) |
| KRT5 (cytoplasm) | 0.57 | (0.29; 0.75) | -0.10 | (-0.41; 0.23) | -0.61 | (-0.78; -0.35) | 0.47 | (0.17; 0.69) |
| MMP2 (cytoplasm) | 0.45 | (0.14; 0.67) | 0.37 | (0.05; 0.62) | -0.10 | (-0.41; 0.24) | 0.44 | (0.13; 0.67) |
| MUC1 (membrane) | 0.10 | (-0.23; 0.41) | 1.00 |  | 0.59 | (0.32; 0.76) | -0.15 | (-0.45; 0.18) |
| MUC1 (cytoplasm) | -0.34 | (-0.59; -0.01) | 0.68 | (0.45; 0.82) | 0.68 | (0.45; 0.82) | -0.40 | (-0.64; -0.08) |
| NKX2-1 (nucleus) | -0.36 | (-0.61; -0.04) | 0.59 | (0.32; 0.76) | 1.00 |  | -0.40 | (-0.64; -0.08) |
| NOTCH1 (cytoplasm) | 0.68 | (0.45; 0.82) | -0.15 | (-0.45; 0.18) | -0.40 | (-0.64; -0.08) | 1.00 |  |
| PAK1 (nucleus) | -0.01 | (-0.33; 0.32) | -0.41 | (-0.64; -0.09) | -0.29 | (-0.56; 0.04) | 0.15 | (-0.19; 0.45) |
| PTGS2 (cytoplasm) | 0.82 | (0.67; 0.90) | -0.10 | (-0.41; 0.24) | -0.50 | (-0.71; -0.20) | 0.65 | (0.41; 0.80) |
| SFTPC (cytoplasm) | 0.79 | (0.62; 0.89) | 0.44 | (0.13; 0.67) | -0.20 | (-0.49; 0.14) | 0.52 | (0.23; 0.72) |
| SNAI1 (nucleus) | 0.33 | (0.00; 0.59) | -0.63 | (-0.79; -0.38) | -0.54 | (-0.73; -0.25) | 0.33 | (0.01; 0.59) |
| SNAI1 (cytoplasm) | 0.18 | (-0.15; 0.48) | -0.07 | (-0.39; 0.26) | -0.07 | (-0.39; 0.26) | 0.28 | (-0.06; 0.55) |
| STAT3 (nucleus) | 0.40 | (0.08; 0.64) | 0.14 | (-0.19; 0.44) | -0.38 | (-0.62; -0.06) | 0.39 | (0.07; 0.63) |
| STAT3 (cytoplasm) | 0.62 | (0.37; 0.79) | 0.14 | (-0.19; 0.44) | -0.37 | (-0.62; -0.05) | 0.60 | (0.33; 0.77) |
| TP53 (nucleus) | 0.45 | (0.14; 0.67) | -0.38 | (-0.63; -0.06) | -0.58 | (-0.76; -0.31) | 0.37 | (0.05; 0.62) |
| TP53 (cytoplasm) | 0.33 | (0.00; 0.59) | -0.14 | (-0.44; 0.19) | -0.25 | (-0.53; 0.08) | 0.34 | (0.01; 0.60) |
| VEGFA (cytoplasm) | 0.71 | (0.49; 0.84) | -0.40 | (-0.64; -0.08) | -0.51 | (-0.71; -0.21) | 0.70 | (0.48; 0.83) |
| VIM (membrane) | 0.53 | (0.24; 0.73) | 0.03 | (-0.30; 0.35) | -0.36 | (-0.61; -0.03) | 0.40 | (0.08; 0.63) |
| VIM (cytoplasm) | 0.42 | (0.10; 0.65) | 0.25 | (-0.09; 0.52) | -0.15 | (-0.45; 0.18) | 0.24 | (-0.09; 0.52) |

**Table S2. Spearman correlation coefficients between marker scores in lung tissue from uranium miners by level of exposure to radon and arsenic (continued)**

|  | **Low radon and high arsenic exposure (N=33)** | | | | | | | |
| --- | --- | --- | --- | --- | --- | --- | --- | --- |
|  | **HIF1A (cytoplasm)** | | **MUC1 (membrane)** | | **NKX2-1 (nucleus)** | | **NOTCH1 (cytoplasm)** | |
| **Marker** | **r_S_** | **95% CI** | **r_S_** | **95% CI** | **r_S_** | **95% CI** | **r_S_** | **95% CI** |
| CCND1 (nucleus) | 0.73 | (0.51; 0.86) | -0.18 | (-0.49; 0.18) | -0.30 | (-0.58; 0.05) | 0.69 | (0.44; 0.83) |
| CCND1 (cytoplasm) | 0.67 | (0.41; 0.82) | -0.40 | (-0.65; -0.06) | -0.47 | (-0.69; -0.14) | 0.76 | (0.56; 0.87) |
| CD44 (membrane) | 0.77 | (0.58; 0.88) | -0.15 | (-0.47; 0.21) | -0.51 | (-0.72; -0.19) | 0.56 | (0.25; 0.75) |
| CD44 (cytoplasm) | 0.76 | (0.56; 0.87) | -0.18 | (-0.49; 0.18) | -0.43 | (-0.67; -0.09) | 0.58 | (0.29; 0.77) |
| CDH1 (membrane) | 0.75 | (0.53; 0.86) | -0.17 | (-0.48; 0.19) | -0.42 | (-0.66; -0.08) | 0.71 | (0.48; 0.84) |
| CDH1 (cytoplasm) | 0.33 | (-0.02; 0.60) | -0.39 | (-0.65; -0.05) | -0.52 | (-0.73; -0.21) | 0.40 | (0.06; 0.65) |
| CTNNB1 (membrane) | 0.70 | (0.46; 0.84) | -0.16 | (-0.47; 0.20) | -0.40 | (-0.65; -0.06) | 0.65 | (0.39; 0.81) |
| CTNNB1 (cytoplasm) | 0.65 | (0.38; 0.81) | -0.07 | (-0.40; 0.28) | -0.10 | (-0.43; 0.25) | 0.64 | (0.37; 0.80) |
| EGFR (membrane) | 0.83 | (0.67; 0.91) | -0.19 | (-0.49; 0.17) | -0.36 | (-0.62; -0.02) | 0.83 | (0.67; 0.91) |
| EGFR (cytoplasm) | 0.71 | (0.47; 0.84) | -0.34 | (-0.61; 0.01) | -0.37 | (-0.63; -0.03) | 0.90 | (0.80; 0.95) |
| ERBB2 (cytoplasm) | 0.59 | (0.29; 0.77) | -0.10 | (-0.43; 0.25) | -0.22 | (-0.52; 0.14) | 0.53 | (0.21; 0.73) |
| HIF1A (cytoplasm) | 1.00 |  | 0.03 | (-0.32; 0.37) | -0.16 | (-0.48; 0.19) | 0.77 | (0.56; 0.88) |
| KIT (cytoplasm) | 0.38 | (0.04; 0.64) | -0.44 | (-0.68; -0.11) | -0.53 | (-0.74; -0.22) | 0.33 | (-0.02; 0.60) |
| KRT14 (membrane) | 0.57 | (0.28; 0.76) | -0.13 | (-0.45; 0.22) | -0.61 | (-0.78; -0.32) | 0.29 | (-0.06; 0.58) |
| KRT14 (cytoplasm) | 0.65 | (0.38; 0.81) | -0.09 | (-0.42; 0.27) | -0.55 | (-0.75; -0.25) | 0.39 | (0.05; 0.65) |
| KRT5 (membrane) | 0.61 | (0.33; 0.79) | -0.15 | (-0.47; 0.20) | -0.55 | (-0.75; -0.25) | 0.40 | (0.05; 0.65) |
| KRT5 (cytoplasm) | 0.69 | (0.45; 0.83) | -0.12 | (-0.44; 0.23) | -0.55 | (-0.75; -0.24) | 0.46 | (0.13; 0.69) |
| MMP2 (cytoplasm) | 0.83 | (0.68; 0.91) | 0.00 | (-0.34; 0.34) | -0.10 | (-0.43; 0.25) | 0.66 | (0.41; 0.82) |
| MUC1 (membrane) | 0.03 | (-0.32; 0.37) | 1.00 |  | 0.71 | (0.48; 0.85) | -0.28 | (-0.56; 0.08) |
| MUC1 (cytoplasm) | -0.08 | (-0.41; 0.27) | 0.66 | (0.41; 0.82) | 0.76 | (0.56; 0.87) | -0.25 | (-0.55; 0.10) |
| NKX2-1 (nucleus) | -0.16 | (-0.48; 0.19) | 0.71 | (0.48; 0.85) | 1.00 |  | -0.22 | (-0.52; 0.14) |
| NOTCH1 (cytoplasm) | 0.77 | (0.56; 0.88) | -0.28 | (-0.56; 0.08) | -0.22 | (-0.52; 0.14) | 1.00 |  |
| PAK1 (nucleus) | 0.23 | (-0.13; 0.53) | -0.48 | (-0.71; -0.16) | -0.45 | (-0.69; -0.12) | 0.34 | (-0.01; 0.61) |
| PTGS2 (cytoplasm) | 0.81 | (0.64; 0.90) | -0.30 | (-0.58; 0.05) | -0.44 | (-0.67; -0.10) | 0.68 | (0.44; 0.83) |
| SFTPC (cytoplasm) | 0.78 | (0.58; 0.88) | 0.33 | (-0.01; 0.60) | 0.14 | (-0.21; 0.46) | 0.56 | (0.26; 0.75) |
| SNAI1 (nucleus) | 0.12 | (-0.23; 0.44) | -0.67 | (-0.82; -0.42) | -0.55 | (-0.75; -0.24) | 0.40 | (0.06; 0.65) |
| SNAI1 (cytoplasm) | 0.43 | (0.10; 0.67) | -0.14 | (-0.46; 0.21) | -0.16 | (-0.48; 0.20) | 0.37 | (0.03; 0.63) |
| STAT3 (nucleus) | 0.40 | (0.06; 0.65) | 0.03 | (-0.32; 0.37) | -0.18 | (-0.49; 0.17) | 0.23 | (-0.13; 0.53) |
| STAT3 (cytoplasm) | 0.55 | (0.25; 0.75) | 0.34 | (-0.01; 0.61) | 0.17 | (-0.19; 0.48) | 0.25 | (-0.10; 0.55) |
| TP53 (nucleus) | 0.13 | (-0.22; 0.45) | -0.44 | (-0.68; -0.11) | -0.49 | (-0.71; -0.17) | 0.25 | (-0.11; 0.54) |
| TP53 (cytoplasm) | 0.32 | (-0.03; 0.59) | -0.25 | (-0.54; 0.11) | -0.25 | (-0.55; 0.10) | 0.37 | (0.02; 0.63) |
| VEGFA (cytoplasm) | 0.72 | (0.49; 0.85) | -0.26 | (-0.55; 0.10) | -0.26 | (-0.55; 0.10) | 0.79 | (0.61; 0.89) |
| VIM (membrane) | No staining | | | | | | | |
| VIM (cytoplasm) | 0.24 | (-0.12; 0.54) | 0.03 | (-0.32; 0.37) | -0.02 | (-0.36; 0.32) | 0.15 | (-0.21; 0.47) |

**Table S2. Spearman correlation coefficients between marker scores in lung tissue from uranium miners by level of exposure to radon and arsenic (continued)**

|  | **Low radon and low arsenic exposure (N=36)** | | | | | | | |
| --- | --- | --- | --- | --- | --- | --- | --- | --- |
|  | **HIF1A (cytoplasm)** | | **MUC1 (membrane)** | | **NKX2-1 (nucleus)** | | **NOTCH1 (cytoplasm)** | |
| **Marker** | **r_S_** | **95% CI** | **r_S_** | **95% CI** | **r_S_** | **95% CI** | **r_S_** | **95% CI** |
| CCND1 (nucleus) | 0.63 | (0.37; 0.79) | -0.09 | (-0.40; 0.25) | -0.17 | (-0.47; 0.17) | 0.65 | (0.41; 0.81) |
| CCND1 (cytoplasm) | 0.78 | (0.60; 0.88) | -0.18 | (-0.48; 0.16) | -0.24 | (-0.53; 0.10) | 0.72 | (0.51; 0.85) |
| CD44 (membrane) | 0.55 | (0.26; 0.74) | 0.03 | (-0.30; 0.35) | -0.45 | (-0.67; -0.14) | 0.53 | (0.24; 0.73) |
| CD44 (cytoplasm) | 0.45 | (0.14; 0.68) | -0.11 | (-0.43; 0.22) | -0.50 | (-0.71; -0.20) | 0.50 | (0.19; 0.71) |
| CDH1 (membrane) | 0.79 | (0.61; 0.88) | -0.04 | (-0.37; 0.29) | -0.24 | (-0.52; 0.10) | 0.72 | (0.51; 0.85) |
| CDH1 (cytoplasm) | 0.73 | (0.52; 0.85) | -0.03 | (-0.35; 0.30) | -0.22 | (-0.51; 0.12) | 0.59 | (0.31; 0.76) |
| CTNNB1 (membrane) | 0.83 | (0.69; 0.91) | -0.03 | (-0.35; 0.30) | -0.27 | (-0.55; 0.07) | 0.82 | (0.67; 0.90) |
| CTNNB1 (cytoplasm) | 0.82 | (0.67; 0.90) | 0.08 | (-0.26; 0.40) | -0.11 | (-0.42; 0.23) | 0.67 | (0.43; 0.82) |
| EGFR (membrane) | 0.74 | (0.54; 0.86) | -0.04 | (-0.36; 0.29) | -0.38 | (-0.63; -0.05) | 0.72 | (0.50; 0.84) |
| EGFR (cytoplasm) | 0.73 | (0.53; 0.85) | -0.11 | (-0.42; 0.23) | -0.36 | (-0.61; -0.03) | 0.69 | (0.46; 0.83) |
| ERBB2 (cytoplasm) | 0.74 | (0.54; 0.86) | -0.02 | (-0.34; 0.31) | -0.18 | (-0.48; 0.16) | 0.70 | (0.48; 0.84) |
| HIF1A (cytoplasm) | 1.00 |  | 0.20 | (-0.14; 0.49) | 0.01 | (-0.32; 0.34) | 0.74 | (0.54; 0.86) |
| KIT (cytoplasm) | 0.46 | (0.15; 0.68) | -0.18 | (-0.48; 0.16) | 0.05 | (-0.29; 0.37) | 0.37 | (0.04; 0.62) |
| KRT14 (membrane) | 0.43 | (0.11; 0.66) | -0.01 | (-0.34; 0.32) | -0.58 | (-0.76; -0.30) | 0.57 | (0.29; 0.75) |
| KRT14 (cytoplasm) | 0.46 | (0.15; 0.68) | 0.01 | (-0.32; 0.34) | -0.49 | (-0.70; -0.19) | 0.62 | (0.36; 0.79) |
| KRT5 (membrane) | 0.48 | (0.17; 0.69) | -0.02 | (-0.35; 0.31) | -0.58 | (-0.76; -0.30) | 0.56 | (0.27; 0.74) |
| KRT5 (cytoplasm) | 0.53 | (0.24; 0.73) | -0.03 | (-0.35; 0.31) | -0.54 | (-0.73; -0.25) | 0.66 | (0.42; 0.81) |
| MMP2 (cytoplasm) | 0.70 | (0.48; 0.83) | 0.01 | (-0.32; 0.34) | -0.07 | (-0.39; 0.26) | 0.55 | (0.27; 0.74) |
| MUC1 (membrane) | 0.20 | (-0.14; 0.49) | 1.00 |  | 0.61 | (0.35; 0.78) | -0.02 | (-0.34; 0.31) |
| MUC1 (cytoplasm) | 0.19 | (-0.15; 0.49) | 0.68 | (0.44; 0.82) | 0.68 | (0.44; 0.82) | -0.03 | (-0.35; 0.30) |
| NKX2-1 (nucleus) | 0.01 | (-0.32; 0.34) | 0.61 | (0.35; 0.78) | 1.00 |  | -0.24 | (-0.53; 0.10) |
| NOTCH1 (cytoplasm) | 0.74 | (0.54; 0.86) | -0.02 | (-0.34; 0.31) | -0.24 | (-0.53; 0.10) | 1.00 |  |
| PAK1 (nucleus) | 0.74 | (0.54; 0.86) | 0.01 | (-0.32; 0.34) | -0.19 | (-0.49; 0.15) | 0.69 | (0.46; 0.83) |
| PTGS2 (cytoplasm) | 0.70 | (0.47; 0.83) | -0.05 | (-0.37; 0.28) | 0.02 | (-0.31; 0.34) | 0.56 | (0.28; 0.75) |
| SFTPC (cytoplasm) | 0.88 | (0.78; 0.94) | 0.30 | (-0.04; 0.57) | 0.02 | (-0.31; 0.34) | 0.71 | (0.48; 0.84) |
| SNAI1 (nucleus) | 0.76 | (0.56; 0.87) | 0.00 | (-0.32; 0.33) | -0.16 | (-0.46; 0.18) | 0.58 | (0.31; 0.76) |
| SNAI1 (cytoplasm) | 0.20 | (-0.14; 0.49) | 0.02 | (-0.31; 0.34) | 0.04 | (-0.29; 0.37) | 0.33 | (0.00; 0.59) |
| STAT3 (nucleus) | -0.01 | (-0.33; 0.32) | 0.25 | (-0.09; 0.53) | 0.21 | (-0.13; 0.50) | -0.07 | (-0.39; 0.27) |
| STAT3 (cytoplasm) | 0.78 | (0.60; 0.88) | 0.33 | (-0.01; 0.59) | 0.32 | (-0.02; 0.58) | 0.63 | (0.37; 0.79) |
| TP53 (nucleus) | 0.47 | (0.16; 0.69) | -0.22 | (-0.51; 0.12) | -0.26 | (-0.54; 0.08) | 0.38 | (0.05; 0.63) |
| TP53 (cytoplasm) | 0.42 | (0.10; 0.66) | 0.05 | (-0.28; 0.37) | 0.42 | (0.11; 0.66) | 0.44 | (0.13; 0.67) |
| VEGFA (cytoplasm) | 0.54 | (0.25; 0.74) | -0.38 | (-0.63; -0.06) | -0.36 | (-0.62; -0.04) | 0.55 | (0.26; 0.74) |
| VIM (membrane) | 0.40 | (0.07; 0.64) | -0.05 | (-0.37; 0.28) | -0.28 | (-0.55; 0.06) | 0.38 | (0.05; 0.63) |
| VIM (cytoplasm) | 0.53 | (0.23; 0.73) | -0.01 | (-0.34; 0.32) | -0.08 | (-0.40; 0.25) | 0.58 | (0.31; 0.76) |
