# Supplementary material for: NOTCH1, HIF1A and Other Cancer-Related Proteins in Lung Tissue from Uranium Miners—Variation by Occupational Exposure and Subtype of Lung Cancer
Source: PLoS One. 2012 Sep 17;7(9):e45305. doi: 10.1371/journal.pone.0045305 (PMC3444449; doi:10.1371/journal.pone.0045305)
Supplement: Table S3 — Proportion of samples with positive staining of candidate proteins in lung cancer and in cancer-free tissue from uranium miners by level of exposure to radon and arsenic. (DOC) [file pone.0045305.s005.doc]

**Table S3. Proportion of samples with positive staining of candidate proteins in lung cancer and in cancer-free tissue from uranium miners by level of exposure to radon and arsenic**

|  | **High radon, high arsenic (N=40)** | | | **High radon, low arsenic (N=37)** | | | **Low radon, high arsenic (N=33)** | | | **Low radon, low arsenic (N=36)** | | |
| --- | --- | --- | --- | --- | --- | --- | --- | --- | --- | --- | --- | --- |
|  | **Membrane** | **Cytoplasm** | **Nucleus** | **Membrane** | **Cytoplasm** | **Nucleus** | **Membrane** | **Cytoplasm** | **Nucleus** | **Membrane** | **Cytoplasm** | **Nucleus** |
| **CCND1** | 1 (3%) | 31 (78%) | 19 (48%) | 2 (5%) | 24 (65%) | 11 (30%) | 0 | 20 (61%) | 13 (39%) | 1 (3%) | 24 (67%) | 13 (36%) |
| **CD44** | 9 (23%) | 10 (25%) | 0 | 12 (32%) | 11 (30%) | 0 | 13 (39%) | 14 (42%) | 0 | 12 (33%) | 14 (39%) | 0 |
| **CDH1** | 22 (55%) | 23 (58%) | 0 | 22 (59%) | 22 (59%) | 0 | 15 (45%) | 18 (55%) | 0 | 21 (58%) | 19 (53%) | 0 |
| **CTNNB1** | 20 (50%) | 33 (83%) | 0 | 18 (49%) | 23 (62%) | 0 | 13 (39%) | 21 (64%) | 1 (3%) | 18 (50%) | 19 (53%) | 0 |
| **EGFR** | 17 (43%) | 20 (50%) | 0 | 16 (43%) | 18 (49%) | 0 | 14 (42%) | 18 (55%) | 0 | 20 (56%) | 22 (61%) | 0 |
| **ERBB2** | 0 | 23 (58%) | 0 | 1 (3%) | 12 (32%) | 0 | 0 | 9 (27%) | 0 | 0 | 14 (39%) | 0 |
| **HIF1A** | 0 | 37 (93%) | 1 (3%) | 0 | 33 (89%) | 1 (3%) | 0 | 27 (82%) | 0 | 0 | 28 (78%) | 1 (3%) |
| **KIT** | 1 (3%) | 21 (53%) | 0 | 0 | 20 (54%) | 0 | 0 | 12 (36%) | 0 | 2 (6%) | 15 (42%) | 0 |
| **KRT5** | 10 (25%) | 12 (30%) | 0 | 11 (30%) | 11 (30%) | 0 | 8 (24%) | 9 (27%) | 0 | 9 (25%) | 11 (31%) | 0 |
| **KRT14** | 4 (10%) | 7 (18%) | 0 | 4 (11%) | 9 (24%) | 0 | 7 (21%) | 8 (24%) | 0 | 9 (25%) | 10 (28%) | 0 |
| **MMP2** | 0 | 21 (53%) | 0 | 0 | 13 (35%) | 0 | 0 | 18 (55%) | 0 | 0 | 17 (47%) | 0 |
| **MUC1** | 29 (73%) | 38 (95%) | 0 | 23 (62%) | 36 (97%) | 0 | 26 (79%) | 33 (100%) | 0 | 26 (72%) | 36 (100%) | 0 |
| **NKX2-1** | 0 | 0 | 29 (73%) | 0 | 0 | 30 (81%) | 0 | 0 | 22 (67%) | 0 | 0 | 22 (61%) |
| **NOTCH1** | 0 | 24 (60%) | 3 (8%) | 0 | 18 (49%) | 1 (3%) | 0 | 17 (52%) | 0 | 0 | 15 (42%) | 0 |
| **PAK1** | 0 | 2 (5%) | 24 (60%) | 0 | 1 (3%) | 13 (35%) | 0 | 5 (15%) | 11 (33%) | 0 | 2 (6%) | 14 (39%) |
| **PTGS2** | 0 | 24 (60%) | 0 | 0 | 17 (46%) | 1 (3%) | 0 | 14 (42%) | 0 | 1 (3%) | 16 (44%) | 0 |
| **SFTPC** | 0 | 34 (85%) | 1 (3%) | 0 | 27 (73%) | 0 | 0 | 25 (76%) | 0 | 0 | 25 (69%) | 0 |
| **SNAI1** | 0 | 9 (23%) | 27 (68%) | 0 | 5 (14%) | 26 (70%) | 0 | 4 (12%) | 22 (67%) | 0 | 2 (6%) | 20 (56%) |
| **STAT3** | 0 | 40 (100%) | 8 (20%) | 0 | 29 (78%) | 7 (19%) | 0 | 23 (70%) | 10 (30%) | 0 | 33 (92%) | 8 (22%) |
| **TP53** | 0 | 3 (8%) | 37 (93%) | 0 | 10 (27%) | 32 (86%) | 0 | 3 (9%) | 27 (82%) | 0 | 4 (11%) | 31 (86%) |
| **VEGFA** | 0 | 30 (75%) | 2 (5%) | 2 (5%) | 28 (76%) | 1 (3%) | 0 | 15 (45%) | 1 (3%) | 0 | 29 (81%) | 3 (8%) |
| **VIM** | 4 (10%) | 10 (25%) | 0 | 6 (16%) | 9 (24%) | 0 | 0 | 6 (18%) | 0 | 4 (11%) | 6 (17%) | 0 |
