# Supplementary material for: NOTCH1, HIF1A and Other Cancer-Related Proteins in Lung Tissue from Uranium Miners—Variation by Occupational Exposure and Subtype of Lung Cancer
Source: PLoS One. 2012 Sep 17;7(9):e45305. doi: 10.1371/journal.pone.0045305 (PMC3444449; doi:10.1371/journal.pone.0045305)
Supplement: Table S4 — Spearman correlation coefficients of marker scores with cumulative exposure to radon [WLM] and arsenic [µg/m3 years] in lung tissue from uranium miners. (DOC) [file pone.0045305.s006.doc]

**Table S4. Spearman correlation coefficients of marker scores with cumulative exposure to radon [WLM] and arsenic [µg/m^3^ years] in lung tissue from uranium miners**

|  | **All samples (N=146)** | | | | **Lung cancer samples (N=107)** | | | |
| --- | --- | --- | --- | --- | --- | --- | --- | --- |
|  | **Radon** | | **Arsenic** | | **Radon** | | **Arsenic** | |
| **Marker** | **r_S_** | **95% CI** | **r_S_** | **95% CI** | **r_S_** | **95% CI** | **r_S_** | **95% CI** |
| CCND1 (cytoplasm) | 0.22 | (0.06;0.37) | 0.12 | (-0.04;0.28) | 0.34 | (0.16;0.49) | 0.18 | (-0.01;0.36) |
| CCND1 (nucleus) | 0.08 | (-0.08;0.24) | 0.10 | (-0.07;0.25) | 0.10 | (-0.09;0.28) | 0.10 | (-0.09;0.28) |
| CD44 (cytoplasm) | -0.11 | (-0.27;0.06) | 0.03 | (-0.14;0.19) | -0.15 | (-0.33;0.05) | 0.02 | (-0.17;0.21) |
| CD44 (membrane) | -0.04 | (-0.20;0.12) | 0.02 | (-0.14;0.18) | -0.06 | (-0.24;0.14) | 0.02 | (-0.17;0.21) |
| CDH1 (cytoplasm) | 0.13 | (-0.03;0.29) | -0.04 | (-0.20;0.12) | 0.18 | (-0.01;0.36) | -0.10 | (-0.28;0.09) |
| CDH1 (membrane) | 0.04 | (-0.12;0.20) | -0.06 | (-0.22;0.11) | 0.04 | (-0.15;0.23) | -0.10 | (-0.28;0.09) |
| CTNNB1 (cytoplasm) | 0.12 | (-0.04;0.28) | 0.11 | (-0.05;0.27) | 0.06 | (-0.13;0.25) | 0.06 | (-0.13;0.25) |
| CTNNB1 (membrane) | 0.09 | (-0.07;0.25) | 0.00 | (-0.16;0.16) | 0.12 | (-0.08;0.30) | -0.01 | (-0.20;0.18) |
| EGFR (cytoplasm) | 0.00 | (-0.16;0.17) | -0.03 | (-0.19;0.14) | 0.00 | (-0.19;0.19) | -0.04 | (-0.23;0.15) |
| EGFR (membrane) | 0.00 | (-0.16;0.16) | -0.01 | (-0.17;0.16) | -0.01 | (-0.20;0.18) | 0.00 | (-0.19;0.19) |
| ERBB2 (cytoplasm) | 0.22 | (0.05;0.36) | 0.18 | (0.02;0.33) | 0.28 | (0.10;0.45) | 0.24 | (0.05;0.41) |
| HIF1A (cytoplasm) | 0.12 | (-0.04;0.28) | 0.03 | (-0.13;0.19) | 0.12 | (-0.07;0.30) | 0.02 | (-0.17;0.21) |
| KIT (cytoplasm) | 0.16 | (-0.01;0.31) | 0.03 | (-0.13;0.19) | 0.20 | (0.01;0.37) | 0.03 | (-0.16;0.22) |
| KRT14 (cytoplasm) | -0.08 | (-0.24;0.08) | -0.07 | (-0.23;0.10) | -0.11 | (-0.30;0.08) | -0.09 | (-0.27;0.10) |
| KRT14 (membrane) | -0.13 | (-0.29;0.03) | -0.05 | (-0.21;0.12) | -0.17 | (-0.35;0.02) | -0.06 | (-0.25;0.13) |
| KRT5 (cytoplasm) | 0.05 | (-0.11;0.21) | 0.04 | (-0.13;0.20) | 0.07 | (-0.12;0.25) | 0.03 | (-0.16;0.22) |
| KRT5 (membrane) | 0.07 | (-0.09;0.23) | 0.01 | (-0.15;0.17) | 0.09 | (-0.11;0.27) | 0.00 | (-0.19;0.19) |
| MMP2 (cytoplasm) | 0.03 | (-0.13;0.20) | 0.12 | (-0.05;0.27) | -0.01 | (-0.20;0.18) | 0.10 | (-0.09;0.29) |
| MUC1 (cytoplasm) | 0.03 | (-0.13;0.19) | 0.00 | (-0.16;0.17) | 0.02 | (-0.17;0.21) | -0.06 | (-0.25;0.13) |
| MUC1 (membrane) | 0.03 | (-0.13;0.19) | 0.00 | (-0.16;0.16) | 0.07 | (-0.12;0.26) | 0.06 | (-0.14;0.24) |
| NKX2-1 (nucleus) | 0.07 | (-0.10;0.23) | 0.01 | (-0.15;0.17) | 0.11 | (-0.08;0.29) | 0.00 | (-0.19;0.19) |
| NOTCH1 (cytoplasm) | 0.18 | (0.02;0.33) | 0.23 | (0.07;0.38) | 0.26 | (0.07;0.42) | 0.34 | (0.16;0.49) |
| PAK1 (nucleus) | 0.18 | (0.01;0.33) | 0.12 | (-0.05;0.27) | 0.22 | (0.03;0.39) | 0.14 | (-0.05;0.32) |
| PTGS2 (cytoplasm) | 0.13 | (-0.03;0.29) | 0.10 | (-0.06;0.26) | 0.17 | (-0.02;0.35) | 0.14 | (-0.06;0.32) |
| SFTPC (cytoplasm) | 0.14 | (-0.03;0.29) | 0.10 | (-0.07;0.26) | 0.15 | (-0.04;0.33) | 0.10 | (-0.09;0.29) |
| SNAI1 (cytoplasm) | 0.16 | (-0.01;0.31) | 0.13 | (-0.03;0.29) | 0.19 | (0.00;0.36) | 0.16 | (-0.03;0.34) |
| SNAI1 (nucleus) | 0.12 | (-0.04;0.28) | -0.02 | (-0.18;0.14) | 0.16 | (-0.03;0.34) | -0.07 | (-0.26;0.12) |
| STAT3 (cytoplasm) | 0.18 | (0.02;0.33) | 0.14 | (-0.03;0.29) | 0.27 | (0.09;0.44) | 0.11 | (-0.08;0.29) |
| STAT3 (nucleus) | -0.04 | (-0.20;0.13) | 0.03 | (-0.13;0.19) | 0.02 | (-0.17;0.21) | 0.07 | (-0.13;0.25) |
| TP53 (cytoplasm) | 0.02 | (-0.14;0.18) | -0.12 | (-0.28;0.04) | 0.01 | (-0.18;0.20) | -0.15 | (-0.33;0.04) |
| TP53 (nucleus) | 0.18 | (0.01;0.33) | 0.03 | (-0.14;0.19) | 0.16 | (-0.03;0.34) | 0.00 | (-0.19;0.19) |
| VEGFA (cytoplasm) | 0.08 | (-0.09;0.24) | -0.19 | (-0.34;-0.03) | 0.08 | (-0.12;0.26) | -0.29 | (-0.46;-0.11) |
| VIM (cytoplasm) | 0.08 | (-0.08;0.24) | 0.10 | (-0.07;0.26) | 0.09 | (-0.10;0.28) | 0.13 | (-0.06;0.31) |
| VIM (membrane) | 0.10 | (-0.07;0.26) | -0.14 | (-0.30;0.02) | 0.11 | (-0.08;0.29) | -0.17 | (-0.35;0.02) |

**Table S4. Spearman correlation coefficients of cumulative exposure to radon [WLM] and arsenic [µg/m^3^ years] with 19 marker proteins in samples of lung tissue (continued)**

|  | **Squamous cell carcinoma (N=33)** | | | | **Adenocarcinoma (N=35)** | | | |
| --- | --- | --- | --- | --- | --- | --- | --- | --- |
|  | **Radon** | | **Arsenic** | | **Radon** | | **Arsenic** | |
| **Marker** | **r_S_** | **95% CI** | **r_S_** | **95% CI** | **r_S_** | **95% CI** | **r_S_** | **95% CI** |
| CCND1 (cytoplasm) | 0.48 | (0.15;0.70) | 0.02 | (-0.32;0.36) | 0.36 | (0.02;0.61) | 0.47 | (0.16;0.69) |
| CCND1 (nucleus) | 0.18 | (-0.18;0.49) | 0.02 | (-0.33;0.36) | -0.01 | (-0.34;0.32) | 0.13 | (-0.21;0.44) |
| CD44 (cytoplasm) | -0.29 | (-0.58;0.06) | 0.04 | (-0.31;0.38) | -0.17 | (-0.48;0.17) | 0.12 | (-0.22;0.44) |
| CD44 (membrane) | -0.12 | (-0.44;0.23) | 0.11 | (-0.24;0.44) | -0.20 | (-0.50;0.14) | -0.09 | (-0.41;0.25) |
| CDH1 (cytoplasm) | 0.25 | (-0.10;0.55) | 0.01 | (-0.34;0.35) | -0.15 | (-0.46;0.20) | -0.50 | (-0.71;-0.19) |
| CDH1 (membrane) | -0.12 | (-0.45;0.23) | -0.01 | (-0.35;0.33) | -0.03 | (-0.36;0.31) | -0.31 | (-0.58;0.03) |
| CTNNB1 (cytoplasm) | -0.15 | (-0.47;0.21) | -0.29 | (-0.57;0.06) | -0.18 | (-0.48;0.17) | 0.22 | (-0.12;0.51) |
| CTNNB1 (membrane) | 0.03 | (-0.32;0.37) | -0.08 | (-0.41;0.27) | 0.30 | (-0.04;0.57) | 0.00 | (-0.34;0.33) |
| EGFR (cytoplasm) | 0.07 | (-0.28;0.40) | -0.23 | (-0.53;0.13) | -0.17 | (-0.47;0.18) | 0.13 | (-0.21;0.45) |
| EGFR (membrane) | 0.03 | (-0.32;0.37) | -0.13 | (-0.46;0.22) | -0.18 | (-0.48;0.17) | 0.15 | (-0.19;0.46) |
| ERBB2 (cytoplasm) | 0.40 | (0.06;0.65) | 0.35 | (0.01;0.62) | 0.16 | (-0.18;0.47) | 0.31 | (-0.03;0.58) |
| HIF1A (cytoplasm) | -0.06 | (-0.40;0.29) | -0.05 | (-0.39;0.30) | 0.13 | (-0.21;0.44) | 0.05 | (-0.29;0.37) |
| KIT (cytoplasm) | 0.15 | (-0.20;0.47) | 0.12 | (-0.23;0.44) | 0.00 | (-0.34;0.33) | 0.01 | (-0.33;0.34) |
| KRT14 (cytoplasm) | -0.55 | (-0.74;-0.24) | -0.22 | (-0.52;0.14) | 0.15 | (-0.19;0.46) | 0.07 | (-0.27;0.39) |
| KRT14 (membrane) | -0.51 | (-0.72;-0.20) | -0.19 | (-0.50;0.17) | 0.15 | (-0.19;0.46) | -0.03 | (-0.36;0.30) |
| KRT5 (cytoplasm) | 0.08 | (-0.28;0.41) | 0.05 | (-0.30;0.38) | 0.03 | (-0.31;0.36) | 0.07 | (-0.27;0.39) |
| KRT5 (membrane) | 0.07 | (-0.28;0.40) | -0.08 | (-0.41;0.27) | 0.06 | (-0.28;0.39) | -0.10 | (-0.42;0.24) |
| MMP2 (cytoplasm) | -0.13 | (-0.45;0.22) | -0.18 | (-0.49;0.18) | 0.02 | (-0.31;0.35) | 0.41 | (0.08;0.65) |
| MUC1 (cytoplasm) | -0.11 | (-0.43;0.25) | -0.13 | (-0.45;0.22) | 0.19 | (-0.15;0.49) | 0.22 | (-0.12;0.51) |
| MUC1 (membrane) | 0.10 | (-0.26;0.42) | -0.03 | (-0.37;0.32) | 0.16 | (-0.18;0.47) | 0.11 | (-0.23;0.43) |
| NKX2-1 (nucleus) | - |  | - |  | -0.07 | (-0.39;0.27) | -0.04 | (-0.36;0.30) |
| NOTCH1 (cytoplasm) | 0.34 | (-0.01;0.61) | 0.25 | (-0.11;0.54) | 0.31 | (-0.03;0.58) | 0.73 | (0.51;0.85) |
| PAK1 (nucleus) | -0.01 | (-0.35;0.34) | -0.04 | (-0.38;0.31) | 0.23 | (-0.12;0.52) | 0.22 | (-0.12;0.52) |
| PTGS2 (cytoplasm) | 0.21 | (-0.15;0.51) | 0.26 | (-0.09;0.55) | 0.20 | (-0.15;0.49) | 0.18 | (-0.16;0.48) |
| SFTPC (cytoplasm) | 0.05 | (-0.30;0.39) | -0.08 | (-0.41;0.27) | 0.25 | (-0.09;0.54) | 0.32 | (-0.02;0.59) |
| SNAI1 (cytoplasm) | 0.30 | (-0.05;0.58) | 0.35 | (0.00;0.61) | 0.07 | (-0.27;0.39) | 0.11 | (-0.24;0.42) |
| SNAI1 (nucleus) | -0.01 | (-0.36;0.33) | -0.21 | (-0.51;0.15) | 0.19 | (-0.16;0.49) | -0.20 | (-0.50;0.14) |
| STAT3 (cytoplasm) | 0.26 | (-0.10;0.55) | -0.03 | (-0.37;0.31) | 0.23 | (-0.11;0.52) | 0.22 | (-0.12;0.52) |
| STAT3 (nucleus) | 0.06 | (-0.29;0.39) | 0.13 | (-0.22;0.46) | -0.15 | (-0.46;0.19) | -0.16 | (-0.47;0.18) |
| TP53 (cytoplasm) | 0.05 | (-0.30;0.39) | 0.00 | (-0.34;0.34) | -0.19 | (-0.49;0.16) | -0.19 | (-0.49;0.15) |
| TP53 (nucleus) | 0.04 | (-0.31;0.38) | -0.15 | (-0.47;0.21) | 0.21 | (-0.13;0.51) | -0.01 | (-0.35;0.32) |
| VEGFA (cytoplasm) | -0.22 | (-0.52;0.14) | -0.51 | (-0.73;-0.20) | 0.13 | (-0.22;0.44) | -0.23 | (-0.52;0.11) |
| VIM (cytoplasm) | -0.08 | (-0.41;0.28) | -0.09 | (-0.42;0.27) | 0.09 | (-0.25;0.41) | 0.24 | (-0.11;0.53) |
| VIM (membrane) | -0.08 | (-0.41;0.27) | -0.35 | (-0.62;0.00) | 0.31 | (-0.03;0.58) | -0.09 | (-0.41;0.25) |

**Table S4. Spearman correlation coefficients of cumulative exposure to radon [WLM] and arsenic [µg/m^3^ years] with 19 marker proteins in samples of lung tissue (continued)**

|  | **Small cell lung cancer (N=39)** | | | | **Cancer-free samples (N=39)** | | | |
| --- | --- | --- | --- | --- | --- | --- | --- | --- |
|  | **Radon** | | **Arsenic** | | **Radon** | | **Arsenic** | |
| **Marker** | **r_S_** | **95% CI** | **r_S_** | **95% CI** | **r_S_** | **95% CI** | **r_S_** | **95% CI** |
| CCND1 (cytoplasm) | 0.39 | (0.08;0.63) | 0.30 | (-0.02;0.56) | 0.09 | (-0.24;0.39) | -0.04 | (-0.35;0.28) |
| CCND1 (nucleus) | -0.06 | (-0.37;0.26) | 0.19 | (-0.14;0.47) | - |  | - |  |
| CD44 (cytoplasm) | -0.35 | (-0.60;-0.04) | -0.15 | (-0.44;0.18) | - |  | - |  |
| CD44 (membrane) | -0.20 | (-0.48;0.13) | 0.09 | (-0.23;0.39) | - |  | - |  |
| CDH1 (cytoplasm) | 0.37 | (0.06;0.61) | 0.19 | (-0.13;0.48) | - |  | - |  |
| CDH1 (membrane) | 0.00 | (-0.32;0.31) | -0.16 | (-0.45;0.17) | - |  | - |  |
| CTNNB1 (cytoplasm) | 0.42 | (0.12;0.65) | 0.24 | (-0.09;0.51) | 0.19 | (-0.13;0.48) | 0.19 | (-0.14;0.48) |
| CTNNB1 (membrane) | -0.14 | (-0.43;0.19) | 0.04 | (-0.28;0.35) | - |  | - |  |
| EGFR (cytoplasm) | -0.19 | (-0.48;0.13) | -0.19 | (-0.47;0.14) | - |  | - |  |
| EGFR (membrane) | -0.09 | (-0.40;0.23) | -0.22 | (-0.50;0.10) | - |  | - |  |
| ERBB2 (cytoplasm) | 0.35 | (0.04;0.60) | 0.15 | (-0.17;0.44) | - |  | - |  |
| HIF1A (cytoplasm) | 0.21 | (-0.11;0.49) | 0.00 | (-0.31;0.32) | 0.10 | (-0.22;0.40) | 0.03 | (-0.29;0.34) |
| KIT (cytoplasm) | 0.39 | (0.07;0.62) | 0.00 | (-0.32;0.31) | - |  | - |  |
| KRT14 (cytoplasm) | -0.04 | (-0.35;0.28) | -0.20 | (-0.48;0.13) | - |  | - |  |
| KRT14 (membrane) | -0.22 | (-0.50;0.11) | -0.06 | (-0.37;0.26) | - |  | - |  |
| KRT5 (cytoplasm) | -0.22 | (-0.50;0.11) | -0.06 | (-0.37;0.26) | - |  | - |  |
| KRT5 (membrane) | -0.22 | (-0.50;0.11) | -0.06 | (-0.37;0.26) | - |  | - |  |
| MMP2 (cytoplasm) | -0.10 | (-0.40;0.23) | -0.04 | (-0.35;0.28) | 0.21 | (-0.12;0.49) | 0.14 | (-0.19;0.43) |
| MUC1 (cytoplasm) | 0.09 | (-0.23;0.39) | -0.34 | (-0.59;-0.02) | 0.14 | (-0.19;0.43) | 0.21 | (-0.12;0.49) |
| MUC1 (membrane) | -0.20 | (-0.48;0.13) | 0.16 | (-0.16;0.45) | 0.12 | (-0.20;0.42) | -0.32 | (-0.57;0.00) |
| NKX2-1 (nucleus) | 0.46 | (0.16;0.67) | 0.14 | (-0.19;0.43) | 0.17 | (-0.16;0.46) | 0.17 | (-0.16;0.46) |
| NOTCH1 (cytoplasm) | 0.22 | (-0.10;0.50) | 0.28 | (-0.05;0.54) | -0.12 | (-0.42;0.20) | -0.12 | (-0.42;0.20) |
| PAK1 (nucleus) | 0.43 | (0.13;0.66) | 0.21 | (-0.12;0.49) | - |  | - |  |
| PTGS2 (cytoplasm) | 0.02 | (-0.30;0.33) | 0.13 | (-0.20;0.42) | - |  | - |  |
| SFTPC (cytoplasm) | 0.24 | (-0.09;0.51) | 0.32 | (0.01;0.58) | 0.12 | (-0.20;0.42) | 0.14 | (-0.18;0.44) |
| SNAI1 (cytoplasm) | 0.16 | (-0.17;0.45) | -0.05 | (-0.36;0.27) | - |  | - |  |
| SNAI1 (nucleus) | 0.29 | (-0.03;0.55) | 0.19 | (-0.14;0.47) | -0.05 | (-0.36;0.27) | -0.05 | (-0.36;0.27) |
| STAT3 (cytoplasm) | 0.33 | (0.01;0.58) | 0.23 | (-0.10;0.50) | -0.32 | (-0.57;0.00) | 0.25 | (-0.08;0.52) |
| STAT3 (nucleus) | 0.17 | (-0.16;0.46) | 0.22 | (-0.11;0.50) | -0.19 | (-0.47;0.14) | -0.08 | (-0.39;0.24) |
| TP53 (cytoplasm) | 0.19 | (-0.13;0.48) | -0.23 | (-0.50;0.10) | - |  | - |  |
| TP53 (nucleus) | 0.18 | (-0.15;0.47) | 0.13 | (-0.19;0.43) | 0.40 | (0.09;0.63) | 0.17 | (-0.15;0.46) |
| VEGFA (cytoplasm) | 0.21 | (-0.11;0.49) | -0.23 | (-0.50;0.10) | 0.06 | (-0.26;0.37) | -0.12 | (-0.42;0.21) |
| VIM (cytoplasm) | 0.25 | (-0.07;0.52) | 0.38 | (0.07;0.62) | 0.04 | (-0.28;0.35) | -0.05 | (-0.36;0.27) |
| VIM (membrane) | - |  | - |  | - |  | - |  |
